# Activity in Inferior Parietal and Medial Prefrontal Cortex Signals the Accumulation of Evidence in a Probability Learning Task

**DOI:** 10.1371/journal.pcbi.1002895

**Published:** 2013-01-31

**Authors:** Mathieu d'Acremont, Eleonora Fornari, Peter Bossaerts

**Affiliations:** 1 Computation and Neural Systems, California Institute of Technology, Pasadena, California, United States of America; 2 Department of Radiology, CHUV and University of Lausanne, Lausanne, Switzerland; University of Oxford, United Kingdom

## Abstract

In an uncertain environment, probabilities are key to predicting future events and making adaptive choices. However, little is known about how humans learn such probabilities and where and how they are encoded in the brain, especially when they concern more than two outcomes. During functional magnetic resonance imaging (fMRI), young adults learned the probabilities of uncertain stimuli through repetitive sampling. Stimuli represented payoffs and participants had to predict their occurrence to maximize their earnings. Choices indicated loss and risk aversion but unbiased estimation of probabilities. BOLD response in medial prefrontal cortex and angular gyri increased linearly with the probability of the currently observed stimulus, untainted by its value. Connectivity analyses during rest and task revealed that these regions belonged to the default mode network. The activation of past outcomes in memory is evoked as a possible mechanism to explain the engagement of the default mode network in probability learning. A BOLD response relating to value was detected only at decision time, mainly in striatum. It is concluded that activity in inferior parietal and medial prefrontal cortex reflects the amount of evidence accumulated in favor of competing and uncertain outcomes.

## Author Summary

In order to make adaptive choices, people need to gather evidence to predict what will happen next. In general, the more frequently an event is observed, the more likely it will occur in the future. Thus the probability of an event is useful to predict its future occurrence. Previous studies have identified regions in the brain that react to rewarding or surprising events, but not to likely events. In the present study, participants had to predict payoffs by observing their repeated occurrence. Functional imaging showed that brain activity in inferior parietal and medial prefrontal cortex increased if the currently observed payoff had been seen many times before. This suggests that these two cortical regions accumulate evidence to predict future events. Further analyses revealed that they belonged to the larger default mode network. This network is involved in introspection and remembering. The inferior parietal and medial prefrontal cortex might thus support the prediction of future events by activating memories of past events.

## Introduction

In an uncertain environment, probabilities are crucial information because they improve prediction of future events. For humans, information about the likelihood of events can be described with abstract symbols, for instance with a verbal sentence or a pie chart. But in many situations, probabilities are learned through experience by observing the occurrence of events [1]. Animals can only learn probabilities through experience as they have no access to language. Thus understanding how information about probabilities is acquired in the brain is a fundamental question in decision neuroscience for both humans and animals.

**Figure 1:**
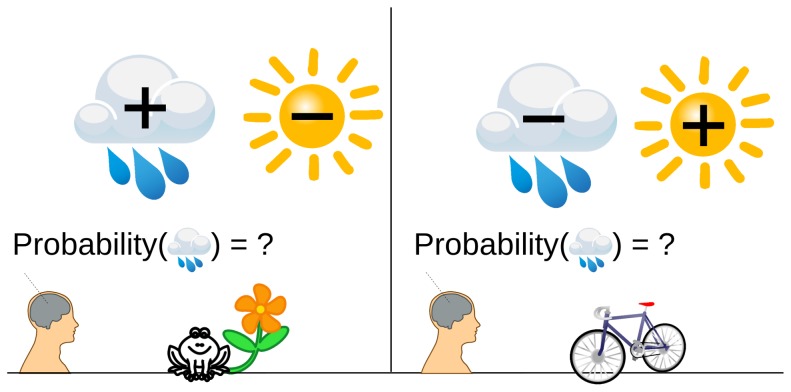
Probabilistic sophistication. A separate estimation of probability and value is necessary to guarantee rational choices in decision theory. This separation also offers more flexibility in goal oriented decisions. Indeed, the subjective values of events change with our goals but not their probabilities of occurrence which are controlled by the environment (or the response of the environment to our actions). For instance, a person is trying to estimate the probability that it will rain. On the left side of the figure, she wants to water her garden. Thus “rain” is a positive event relative to her goal. On the right side, she wants to go for a bike ride. Thus “rain” is a negative event. It can be seen that the subjective value of “rain” changes with personal goals but not the chance it will rain. Therefore, it would be adaptive for the brain to encode the probabilities and values of events separately.

In the present study, we focused on the probability of events independently of their value. The motivation came from the observation that people can memorize, make predictions, and decide in the absence of immediate reinforcements. This ability to build a representation of the environment independently of the rewards to be received is made explicit in model-based reinforcement learning [2]. In addition, a separate estimation of probability and value is necessary to ensure rational choices [3], [4]. This principle called “probabilistic sophistication” might seem counter-intuitive because probabilities are combined with values to estimate expected value in many decision models (e.g., expected utility). Nevertheless, the fact that probabilities and values are multiplied does not contradict the necessity to estimate them independently. The concept of probabilistic sophistication is illustrated in Fig. 1.

In psychology, there has been a long tradition of research showing that people can learn the probabilities of stimuli with no value like neutral words or symbols [5]–[7]. In neuroscience, this type of inference has been studied with categorization tasks [8]. In the weather prediction task for instance, participants have to predict the occurrence of two probabilistic outcomes through trial and error (e.g., sunshine or rain). The probability of the outcome is conditional on a set of four symbols. When comparing this task to a control condition, authors have found activation in a large network including the medial and lateral prefrontal cortex, inferior parietal cortex, posterior cingulate cortex and striatum [9]–[11]. A limitation of these studies is the use of a subtraction instead of a parametric approach. It is thus unknown if regions in the brain encode the probability of the outcome in this task.

Following a parametric approach, authors have observed a larger BOLD response in striatum and ventro medial prefrontal cortex when the probability of an anticipated reward increased [12]–[14]. These results have been interpreted in terms of value because for a single and uncertain reward, probability and expected value are positively correlated. Other regions of the brain, like the parietal cortex and the amygdala, have been found to increase with the probability of an upcoming punishment [15], [16]. To support probabilistic sophistication however, one has to identify structures in the brain which encode probability independently of value.

The effect of value can be controlled for by relating brain activity to the probabilities of events and making sure these probabilities do not covary with reward expectation. An event can be defined as a stimulus, its omission, a feed-back and so on. In reinforcement learning studies, authors have shown a larger BOLD response to the occurrence of rare events [17]–[19]. Activity has generally been found in the lateral parietal and prefrontal cortex. Using EEG, numerous studies have shown an enhanced brain response (P300) to rare target in the odd ball paradigm [20], [21]. It should be noted that in these fMRI and EEG studies, brain activity was not always related to the probability of the outcome, but to other measures like surprise or “state” prediction error (one minus the estimated probability of the outcome). However, these measures are highly and negatively correlated with probability. If the surprise or state prediction error is large, the outcome probability is low.

The brain response to rare events has been explained by associative learning theory (as a prediction error) [18], [19] or statistical inference (as a Bayesian surprise) [17], [21], [22]. In a learning context however, we are not aware of experiments showing a positive correlation between brain activity and event probabilities. This is surprising because several models explain choices as the result of evidence accumulation [23], [24]. When the environment is stable (probabilities do not change overtime), the past occurrence of an uncertain stimulus constitutes evidence for its future occurrence.

In a perceptual decision-making task, the agent has to make a decision based on a noisy signal. Several studies in monkeys have shown that the firing rate of neurons in the lateral intraparietal cortex increased over time as a function of the proportion of dots moving in the same direction [25], [26] and this pattern is well explained with artificial neural networks [27]. In these studies, accumulation of evidence is observed by recording the firing rates of neurons with a specific response field [26], [28]. With fMRI, the researcher only has access to the activation of a large population of neurons and evidence accumulated by neurons of one response field (e.g left direction) might cancel out the evidence accumulated by neurons sensitive to a different response field (e.g. right direction).

In a probability learning task evidence is not presented simultaneously but one after another. This offers the opportunity to relate brain activity to the characteristic of the currently observed evidence (which serves as a probe). If the evidence has been observed many times, retrieval models based on accumulation processes predict a stronger brain response because the probe matches numerous traces of past outcomes in memory [29], [30]. In neuroscience, it has been proposed that the inferior parietal cortex plays the role of a mnemonic accumulator because this area is more activated during the successful recognition of old versus new items [31]–[33]. Other regions of the default mode network (medial temporal lobes, medial prefrontal cortex, posterior cingulate cortex) have been found to be more activated for objects which are easily associated to a specific context compared to objects eliciting weak association [34], [35]. According to the principle of an accumulation of evidence in memory, a positive BOLD response can be expected for likely events, particularly in the default mode network.

Overall, studies have identified regions in the brain where activity increased with the probability of a single and random reward. BOLD response related to reward probability has been observed, mainly in striatum [12], [36] and ventro medial prefrontal cortex [13], [37]. However, when the effect of value was controlled for, an increase of activity in response to unlikely outcomes has been found in lateral parietal and lateral prefrontal cortex [17]–[19]. As such, previous studies on learning have shown that the brain reacts to rewarding or rare events but not to likely events. This conflicts with models of perception and memory [27], [29] where activity increases with the accumulation of evidence. In a probability learning task, we found that activity in bilateral inferior parietal and medial prefrontal cortex increased for events which had been observed many times and were likely to occur again.

## Results

### Rational of the task

**Figure 2:**
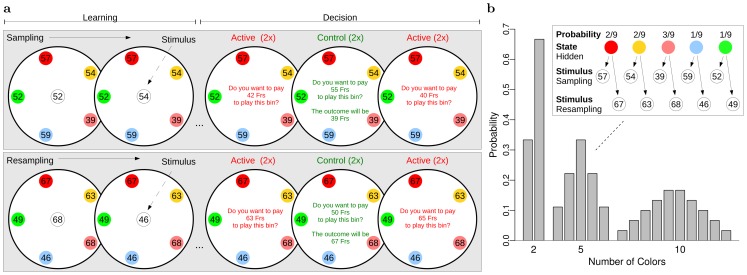
Task design. (**a**) Payoffs were determined by the colors of balls drawn from a bin. In two sampling stages, participants had the opportunity to learn probabilities by observing several drawings. Payoffs were shown in the center of the screen (stimuli). Colors were not displayed (hidden states). After each sampling stage, participants had to decide to buy the gamble or not for a certain price. In the initial sampling stage, both the composition of the bin and the color-payoff association were new. In the resampling stage, the composition of the bin remained the same (same color probabilities) but the associated payoffs were new. (**b**) **Top insert.** The color-payoff association changed in the resampling stage. **Histograms.** These are the true probabilities used to generate the drawings for bins with 2, 5, or 10 colors.

We developed a task where evidence for future outcomes were balls drawn from a bin. The bin contained balls of different colors and each color was associated to a payoff (Fig. 2a). The composition of the bin was hidden, therefore payoff probabilities were unknown to the participants. However, they had the opportunity to learn these probabilities by observing 10 to 14 drawings from the bin. Balls were sampled one after another with replacement and shown in the center of the bin. The sample payoff was displayed but not the color of the ball. Thus colors were hidden states and payoffs were stimuli (Top insert, Fig. 2b). Colors could be inferred from payoffs because the color-payoff association was shown to the participants in the periphery of the bin. After the 10 to 14 draws, this association changed while color probabilities remained constant. In this resampling phase, 10 to 14 balls were drawn again.

At the end of the sampling and resampling phases, participants had to decide to buy or not a gamble for a certain price. After their choice, the payoff was determined by drawing an additional ball from the bin. If participants decided to buy, they earned the price minus the payoff (this net payoff could be negative). If they decided to pass, the net payoff was 0. To maximize their earning, it was optimal for them to predict the payoffs (stimuli) based on the colors (hidden states). Participants learned the probability of 2, 5, or 10 payoffs (Histograms, Fig. 2b).

For the main analysis, brain activity in the sampling stages was regressed on the probability of the currently observed stimulus, that is the probability of seeing the evidence. Probabilities of stimuli were orthogonal to their values and the value to be expected at the end of the sampling or resampling period. For instance, if a red ball was associated with a low payoff, sampling a red ball increased the probability of seeing this low payoff, but it decreased the expected payoff. The independence between probability and value was obtained by randomly assigning payoffs to colors.

### Behavioral choice

To decide whether to buy the gamble for a certain price or to pass it, participants had to predict the gamble payoff at the end of each sampling stage. Analysis was conducted to determine from gamble choices whether participants estimated probabilities based on the color or payoff history (Fig. S1 in Text S1). If inference is based on colors (hidden states), it can be concluded that people are able to make abstraction of rewards when estimating the likelihood of future outcomes. When extracting probabilities from choices, one needs to control for attitudes towards uncertainty. We did so in a generally accepted way, using prospect theory, which allows one to separately control for loss aversion (“losses loom larger than gains”) and differential risk attitudes.

When a new bin was introduced, initial beliefs were set to equiprobable priors, and subsequent updating was assumed to follow Bayes' law. The posterior stimulus probability increased with the accumulation of evidence. At the end of each sampling stage, probabilities and payoff magnitudes (net of the price) were combined to compute gamble expected value according to prospect theory principles. The decision to buy was predicted from valuation with a logistic regression. We compared models with the Bayesian Information Criterion (BIC), because it can be used with non-nested models and limits the risk of over-fit by penalizing free parameters (a lower BIC is better). Models are indexed by the number of free parameter (M2, M3, etc.) and are presented in the *Methods* section below.

The most efficient model (the best compromise between parsimony and fitting) was a model with payoff probabilities calculated conditional on the colors of the balls drawn since the presentation of a new bin (model M4, BIC = 1250, Table 1). Colors were hidden states but could be inferred from the observed payoffs. It was more efficient than a model with payoff probabilities calculated conditional on the payoffs observed since the beginning of the sampling or resampling stage (M4a, BIC = 1271). In this model, participants ignore colors and have to re-estimate probabilities after the payoff-color association changed. Further analyses at the individual level showed that model M4 offered a better fit than M4a for all participants (section *Behavioral choice* & Fig. S2 in Text S1). Thus all participants appeared to be “sophisticated”: their inference was indirect, based on the hidden states behind the observed payoffs, rather than on the observed payoffs directly. Model M4 was more efficient than a model which did not update payoff probabilities (M4b, BIC = 1354). In this latter model, participants used equiprobable payoffs to make decisions (absence of learning).

**Table 1:** Choice models.

Probability Model	*Value*	*Probability*	*Inference*	*BIC*	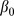	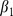	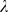	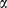	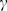
M2	Linear	Linear	Hidden states	1312	−0.49	0.38	-	-	-
M4*	Non-linear	Linear	Hidden states	1250	0.95	0.54	2.57	0.69	-
M4a	Non-linear	Linear	Observations	1271	1.00	0.53	2.66	0.68	-
M4b	Non-linear	Linear	No learning	1354	0.51	1.31	1.69	0.44	-
M5	Non-linear	Non-linear	Hidden states	1251	1.00	0.59	2.55	0.63	0.89
**Reinforcement Model**	***Value***	***Probability***	***Inference***	***BIC***	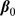	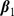	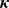		
M3	Linear	Ignored	Observations	1428	−0.39	0.31	0.17	-	-

* Best efficiency; Nbr. data = 1648; 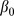 = intercept, 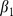 = slope, 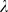 = loss aversion, 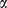 = diminishing sensitivity, 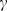 = probability weighting, 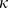 = learning rate; Tversky et al., 1992: 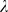 = 2.25, 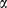 = 0.88/0.88 (gain/loss), 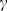 = 0.61/0.69; Hau et al., 08: 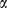 = 0.94/0.86, 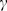 = 0.99/0.93.

**Figure 3:**
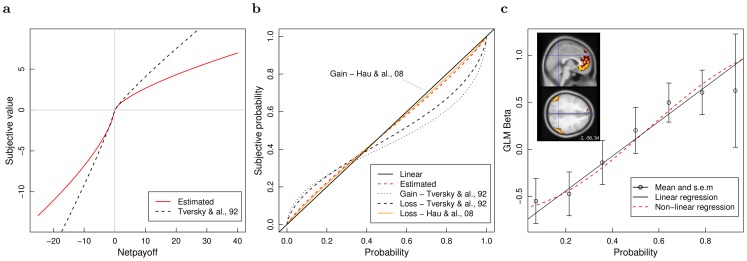
Value and probability functions. (**a**) Value function as estimated from participants' decisions (red, model M4.) Estimation obtained by Tversky and Kahneman (1992) in a study on decisions from description. (**b**) Probability weighting inferred from choices (red, model M5) and comparison with estimations from other studies in the gain and loss domains (Gain - Hau et al., 2008 is confounded with the linear function.) (**c**) **Top insert**. To avoid circularity, ROIs for each individual were determined based on the data of all other participants. ROI voxels common to all participants are shown in yellow. ROI voxels belonging to at least one participant are shown in red. This representation shows to which extent the ROI definitions vary in the cross-validation. **Main**. Increase of BOLD response with stimulus probabilities in medial prefrontal cortex and angular gyri during the learning phase. Data of the 3 ROIs has been merged. The y axis indicates the effect the presentation of new stimulus (payoff) had in the ROIs. The effect increased with the probability of the currently observed stimulus. The non-linear regression (red) includes a probability weighting function.

Model M4 included a prospect theory value function. It was more efficient than a simpler model using a linear value function (M2, BIC = 1312). The shape of the estimated non-linear function revealed diminishing sensitivity for large payoffs (either positive or negative) and greater importance of losses compared to gains. Decision parameters found in previous studies are reported in the footnote of Table 1. Loss aversion was close to the estimation by Tversky and Kahneman in a study made on decisions from description [38]. Diminishing sensitivity was more pronounced in the present study (Fig. 3a).

Model M4 was more efficient than a model that included a prospect theory probability weighting function (M5, BIC = 1251). Indeed, the later model led to a quasi-linear function. We also report the estimation found in decisions from description by Tversky and Kahneman [38] and in decisions from experience by Hau et al. [39]. Probability weighting appears to be minimal in decisions from experience (Fig. 3b).

Finally a reinforcement learning algorithm was estimated (M3). The first payoff observed at the beginning of the sampling or resampling period defined the initial forecast. Then each new payoff was compared to the previous forecast to compute a prediction error. This prediction error multiplied by a learning rate was added to the previous forecast to find the new forecast. This model used a linear value function and bypassed probabilities in order to directly estimate the expected payoff of the gamble. Results showed it had the lowest efficiency (BIC = 1428). Thus probability-based models offered a better explanation of decisions than a reinforcement algorithm.

In sum, the analysis of choices indicated that participants were loss and risk averse (non-linear value function), but there was no indication of a distortion of probabilities (linear probability weighting function). Supporting the principle of probabilistic sophistication, participants learned probabilities based on the hidden states and not simply the observed payoffs. A reinforcement learning model tracking payoffs to estimate gamble values performed worse than any of the probability-based models.

### Brain response to stimulus probabilities

The threshold for significance was set at 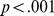, uncorrected, cluster 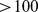 voxels for voxel-based analyses (including the identification of ROIs). False Discovery Rates (FDRs) are reported in tables, to gauge the risk of false-positive results. Coordinates are given in MNI space [mm]. The threshold was set to 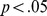 to analyze mean activation in ROIs. Circularity in ROI analysis was avoided with cross-validation. Subject variability was modeled as a random factor in voxel-based and ROI analyses. Details on the GLMs (GLM1, GLM2, etc.) are given in the *Brain analysis* section of Text S1.

The display of a new payoff in the center of the bin is referred as a stimulus and gives evidence for future outcomes. Stimuli were defined as 1 [s] events and led to a significant activation in the occipital cortex and bilateral hippocampus (GLM1, Table S1 in Text S1).

Probabilities inferred from the history of the hidden states were entered as a covariate to modulate the effect of stimuli (model M4). For instance in Fig. 2a (Resampling), when “68” was displayed the probability of seeing “68” was used as a parametric covariate. When “46” was displayed the probability of seeing “46” was used instead. Thus analyses were conducted with the probability of the currently observed stimulus. Probabilities estimated with model M4 ranged from 0.08 to 0.92. The probability of the stimulus did not correlate with its associated payoff magnitude (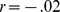, 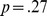) or the gamble expected payoff (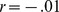, 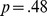). This is because payoffs were randomly assigned to colors which in turn were randomly assigned to probabilities. For example, in Fig. 2a (Resampling), the stimulus “46”, which was the lowest payoff in the bin, could have a low or high probability of occurrence because it was randomly assigned to the color blue. There is thus no confound between probability and value in our design.

**Figure 4:**
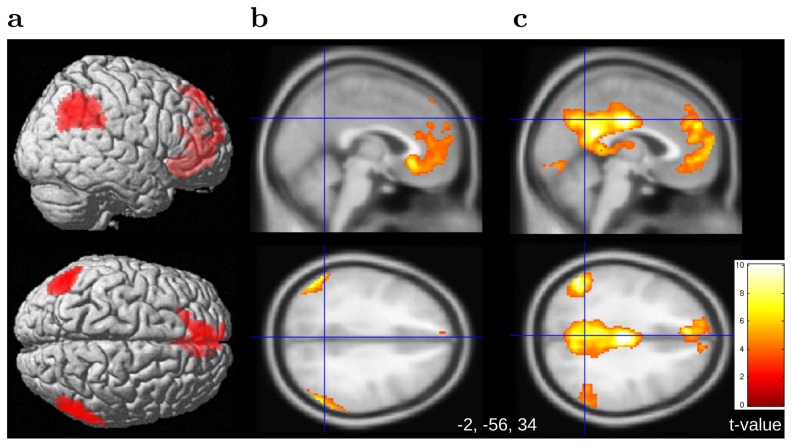
Learning phase. (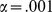, uncorr.) Volume (**a**) and sectional (**b**) views of the BOLD response to stimulus probabilities in medial prefrontal cortex and bilateral angular gyrus. Activity increases with the probability of the currently observed stimulus. (**c**) Voxels showing increased connectivity with angular gyri and medial prefrontal cortex ROIs compared to the resting phase.

Results showed a positive and significant effect of stimulus probabilities medially in the prefrontal cortex and bilaterally in the angular gyrus (Fig. 4a+b, Table S2 in Text S1). In these regions, brain activity increased when a stimulus was likely to be observed (confirmatory signal). A negative and significant effect of probabilities was also observed in the occipital, superior parietal, and middle frontal gyrus. Activity in the middle frontal gyrus was posterior and reached the precentral gyrus (Fig. S3a in Text S1). A significant effect was also observed in the bilateral hippocampus (Table S3 in Text S1). In these regions, brain activity increased when a stimulus – learned to be rare – was observed (surprise signal). Activation related to stimulus probabilities survived correction for multiple comparisons, except in the hippocampus (FWE, 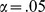).

When a payoff was displayed in the sampling or resampling period, it generated a prediction error. This prediction error was calculated with model M4 as the change in expected value before and after the new payoff was revealed. Results indicated that prediction errors did not increase or decrease the effect of stimuli on the brain (GLM2, no table or figure was reported for this non-significant covariate). Thus, the brain encoded stimulus probabilities but not values during the learning phase. We used the sign of the prediction error to define positive and negative stimuli (GLM3). Results showed BOLD response to probabilities in the angular gyri and medial prefrontal cortex for both positive and negative stimuli (Tables S4 & S5 in Text S1). Thus, the encoding of probabilities was comparable for positive and negative stimuli. In short, it appeared that during the learning phase the brain ignored values (probabilistic sophistication) and focused on probabilities.

A BOLD response to unlikely stimuli has been reported in the literature [17], [19]. The BOLD response to likely stimuli is novel. We will focus on this positive correlation in the rest of the results. ROIs were defined as the cluster of voxel significantly activated for a given variable of interest (GLM4) and mean activations in ROIs (GLM5) were further analyzed with mixed effect regressions. ROIs analyses revealed that BOLD response in angular gyrus and medial prefrontal cortex was better explained by stimulus probabilities inferred from the hidden states (model M4) rather than the observed payoffs (model M4a), in line with the behavioral results. The interaction of probabilities with ROI location was not significant. The effect of stimulus probabilities is thus similar in the three ROIs (Table S6 in Text S1, see [40] for the necessity to test interactions before making simple contrasts). The number of colors did not interact with stimulus probabilities, meaning that the effect of probabilities was not influenced by the number of states (Table S6 in Text S1). When the effect of probabilities was estimated for each number of states, it was found to be significant for 2 (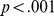), 5 (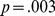), and 10 states (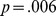, Table S7 in Text S1).

Analysis of choices revealed that expected utility was linear relative to probabilities. We also tested whether BOLD responses in the three ROIs increased linearly with probabilities. The first model included only an intercept and yielded to a BIC of 78707. In the second model, we added a linear effect for stimulus probabilities. This linear effect of probabilities was significant (Table S8 in Text S1) and the BIC fell to 78491, showing an increase in efficiency. Finally, a non linear weighting function was added. The linear effect was again significant (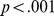). In contrast with the inverted-S shape of prospect theory, there was a slight diminution of sensitivity for probabilities close to 0 and 1 (Fig. 3c) but this non-linear effect was not significant (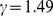, 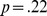, Table S9 in Text S1). The BIC of this model was 78542, showing a decrease in efficiency compared to the previous model. Similar results were found when the non-linear model was tested on each ROI separately (no table was reported for the separate analyses). Thus, we found no evidence for a non-linear encoding of stimulus probabilities when learned from experience.

Connectivity analysis was conducted to explore the functional link between the ROIs encoding stimulus probabilities and the rest of the brain. Each of the ROIs was taken as a seed region in three separate analyses. Results showed that during the learning phase (compared to the resting phase) the correlation increased between each ROI and voxels in the two other ROIs. Correlations also increased between each ROI and the posterior cingulate (no table or figure was reported for the separate analyses). Connections with posterior cingulate cortex were also observed when voxels of the three ROIs were merged to define a single seed region (Fig. 4c, GLM6, Table S10 in Text S1).

### Brain response to value and uncertainty

Comparing the active phase (when choices were made without knowing the outcome in advance) to the control phase (when the outcome was known before making choices), significant activity was observed in the occipital cortex, suggesting that visual exploration of the bin was more intense when the outcome was unknown (GLM1, Table S11 in Text S1). In addition, BOLD responses in the right anterior insula and bilateral caudate were significant. These regions have been involved in risky decision-making, which is present in the active phase but not in the control phase [41].

During the learning phase, we have reported above how brain activity changed as a function of probabilities of a specific stimulus. This approach was possible because stimuli (payoffs) were presented one at a time. But the approach cannot be used when the participants deliberated on their choice because all possible outcomes should be contemplated at once. To study the link between brain activity and probabilities during the decision epoch, a measure that summarizes the set of outcome probabilities should be used instead. Here, we chose entropy, which measures the uncertainty reflected in a set of probabilities. Entropy increases as the probability distribution approaches the uniform distribution.

**Figure 5:**
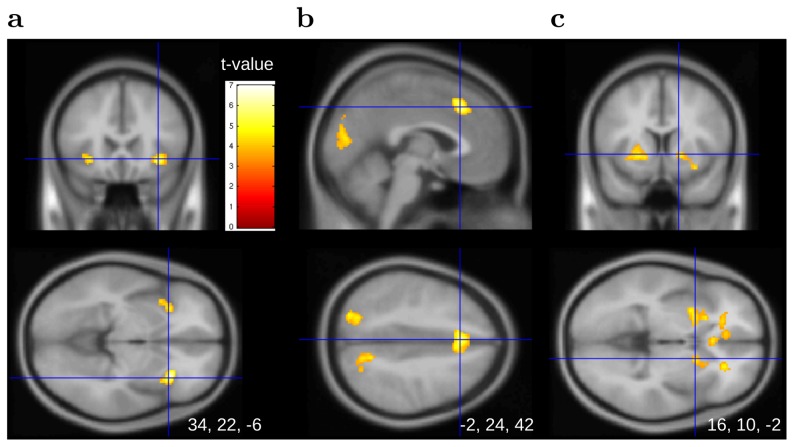
Active decision phase. (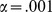, uncorr.) (**a**) BOLD response in the bilateral insula to gambles combining high outcome entropy and high expected value. Outcome uncertainty increased with entropy. (**b**) BOLD response to choice entropy in the dorsal anterior cingulate. Participants faced a more difficult choice when the probabilities to buy and pass the gamble were close, that is when choice entropy was high. (**c**) Striatal activation related to the magnitude of the total payoff received at the end of the decision phase (net of the total price). Whereas activation related to outcome and choice entropy were observed before participants made a choice (anticipation), BOLD response to the net payoff was observed afterwards (feedback).

During the deliberation preceding the decision to buy or pass, the expected gamble value (net of the price) was related to activity in the caudate and spread to other regions in the brain (Fig. S4a, Table S12 in Text S1). At the same period, expected value interacted with outcome entropy in the bilateral insula (Fig. 5a & Table S13 in Text S1). An ROI analysis revealed a main and positive effect of expected value in insula. The interaction showed that this effect of value was stronger when the outcome entropy was high (Fig. S4b+c & Table S14 in Text S1). Thus, the insula seems to be especially sensitive to the value of gambles with uncertain outcomes.

In order to quantify uncertainty regarding choices, we computed the entropy of the probabilities (and complementary probabilities) that participants bought into the gamble (as predicted by model M4). Voxel-based analyses showed a significant effect of choice entropy in dorsal anterior cingulate cortex (Fig. 5b & Table S15 in Text S1). ROI analyses were conducted to further test the double dissociation between outcome and choice entropy in insula and anterior cingulate. Results indicated that choice entropy was specifically encoded in anterior cingulate and not insula. The dissociation was not significant for outcome entropy. Finally, BOLD response in the bilateral striatum (putamen and caudate) was related to the net payoff revealed at the end of each decision phase (Fig. 5c & Table S16 in Text S1).

### Resting phase

The three ROIs found to encode stimulus probabilities along with the posterior cingulate are all key regions of the default mode network. Regions forming the default network have two characteristics: (1) their spontaneous activity is correlated when people are at rest, (2) they are deactivated during tasks requiring attention to external stimuli [42]. The default mode network includes the inferior parietal cortex, the posterior cingulate cortex, the medial prefrontal cortex, the lateral temporal cortex, and the hippocampus.

**Figure 6:**
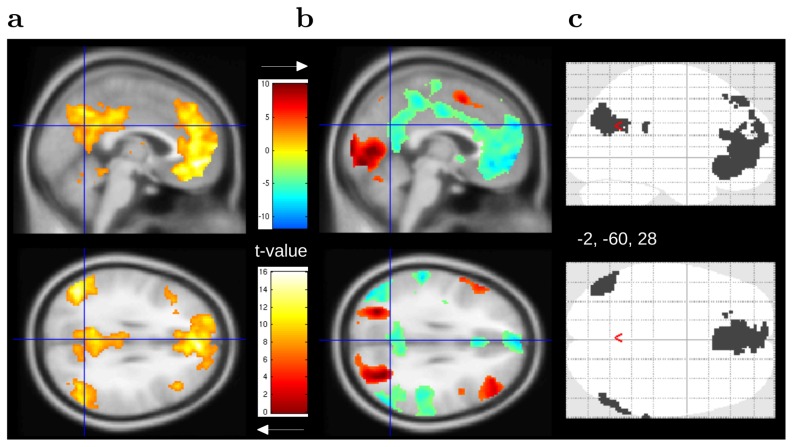
Default mode network. (**a**) Voxels showing connectivity with angular gyri and medial prefrontal cortex ROIs during the resting phase. (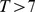, uncorr.) (**b**) Voxels showing activation (red, task-positive network) and deactivation (blue, task-negative network) during the learning and decision phases (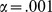, uncorr.) (**c**) Overlap between voxels encoding stimulus probabilities (Fig. 4a+b) and the task-negative network (panel b, blue voxels).

To test the involvement of the default network in the present study, we explored the spontaneous correlations between ROIs encoding probabilities and the whole brain during the resting phase (threshold 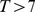). Results showed a functional link between each ROI and voxels in the other two. Each ROI was also connected to activity in the posterior cingulate cortex (no table or figure was reported for the separate analyses). Connections with the posterior cingulate cortex were also observed when voxels of the three ROIs were merged to define a single seed region (Fig. 6a, GLM7, Table S17 in Text S1).

**Figure 7:**
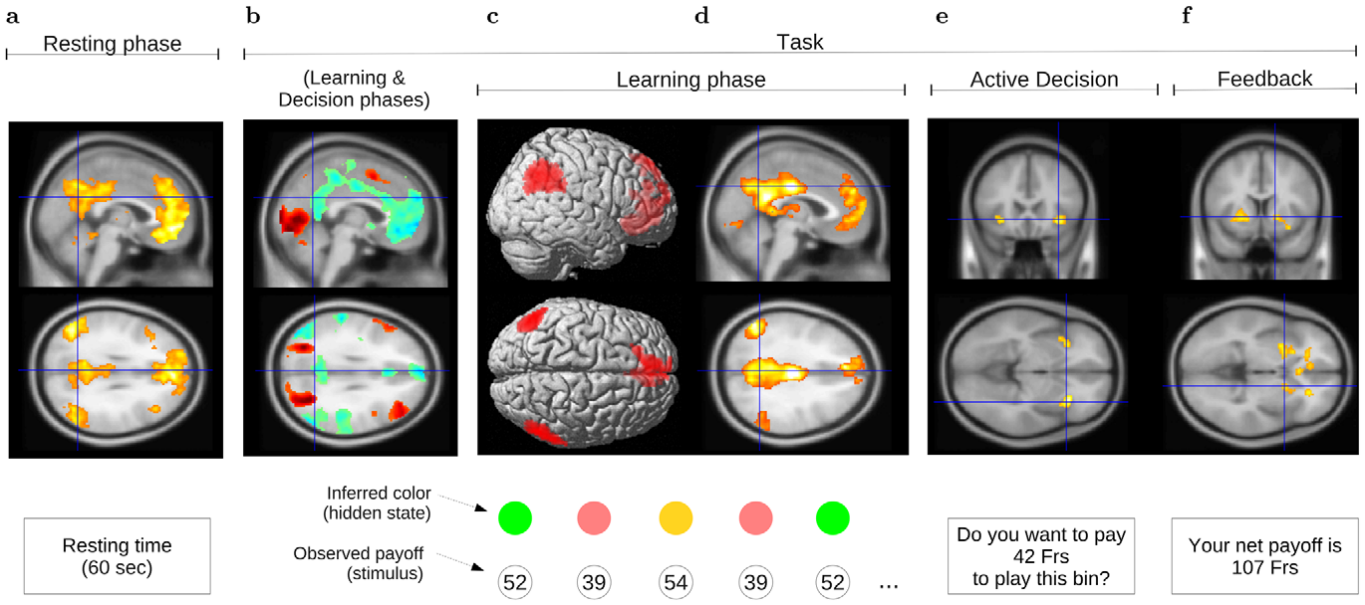
Main findings. (**a**) During the resting phase, the spontaneous activity of the brain correlated between angular gyri, medial prefrontal cortex, and posterior cingulate cortex. This constitutes the first characteristic of the default mode network. (**b**) During the task (learning & decision phases), baseline activity in these regions decreased. This is the second characteristic of the default network. At the same time, activation in the occipital, superior parietal, lateral prefrontal cortex, and other regions involved in visual attention increased. (**c**) During the learning phase, participants only saw the payoff in the center of the bin (stimulus). Nevertheless, the brain encoded the probability of currently observed stimulus inferred from the hidden states (colors). BOLD response for frequent stimuli increased in angular gyri and medial prefrontal cortex. BOLD response for rare stimuli increased in occipital areas, superior parietal cortex, middle frontal gyri, and hippocampus (Fig. S3a in Text S1). (**d**) Compared to the resting phase, correlations between these regions increased during learning. (**e**) When participants had to decide whether to buy the gamble or not, BOLD response in the insula increased with with gamble expected value, especially for when outcome entropy was high. At the same time, the dorsal anterior cingulate cortex signaled choice entropy (Fig. 5b). (**f**) After six choices, a feedback was displayed. The bilateral striatum encoded the net payoff magnitude.

Baseline activity was compared between the decision-making task (learning and decision phases) and the resting phase. Results showed an increase of BOLD response in occipital, superior parietal cortex, supplementary motor areas, and lateral prefrontal cortex (red voxels, Fig. 6b, GLM1, S18 in Text S1). Decreased activity was found bilaterally in angular gyrus, supramarginal gyrus, and middle and superior temporal gyri. Decreased activity was also observed in cingulate and medial prefrontal cortex (blue voxels, Fig. 6b, Table S19 in Text S1, similar results were found when comparing the resting phase to the learning phase only). There was a substantial overlap between the task-negative network and voxels encoding stimulus probabilities during the learning period (Fig. 6c). The results indicated that regions reacting to likely events belonged to the default network. On the other hand, there was a substantial overlap between the task-positive network and voxels reacting to rare stimuli (Fig. S3b in Text S1). For a schematic of the main findings, see Fig. 7.

## Discussion

In a complex and uncertain environment, probabilities are essential for predicting future events. To make consistent choices, it is necessary for a decision maker to separate the chances of objective events (“it will snow”) from the values that could potentially be attached to those events (“we can go skiing”). With such a strategy the decision maker can make inference in the absence of immediate reinforcements and quickly adjust his predictions when the reinforcing values of events change [43]. Here, we developed a paradigm in which the probability and value of stimuli were statistically independent. This allowed us to identify the regions in the brain encoding event probabilities and exclude a confound with value.

Analysis of brain activity during the learning period revealed both positive and negative correlations with stimulus probabilities. BOLD response in angular gyrus and medial prefrontal cortex increased for stimuli which had been observed many times in the current trial. This relationship was significant in conditions with 2, 5, and 10 different stimuli. This shows that the brain can keep track of the probabilities of multiple events. Comparison with the resting state condition and connectivity analyses indicated that these regions belonged to the default mode network and that their baseline activity decreased during the task (task-negative network). A negative correlation between stimulus probabilities was observed in the occipital, superior parietal and lateral prefrontal cortex. Here BOLD response increased for improbable stimuli. These regions were more activated during the task (task-positive network). Before a choice was made activity in striatum and insula increased with gamble expected value. The effect of value in insula was stronger when outcome entropy was high, that is when the future was uncertain. Choice entropy which reflects decision uncertainty was preferentially associated to a BOLD response in dorsal anterior cingulate. After the decision, activity in the striatum increased with the net payoff.

The principle of a separation between probability and value, namely probabilistic sophistication [3], [4], was supported by several results. In the learning period, the effect of probabilities was significant for both positive and negative events and the main effect of probability did not interact with event value. No significant effect of value was observed during the learning period. These results suggest that when reinforcements are delayed, the brain focuses on event probabilities while abstracting from rewards. It is only during the decision period that activation in relation with value was observed.

These results are relevant for the debate on value-based and model-based reinforcement learning [18]. In value-based reinforcement learning, the agent learns the expected value associated to a situation or action by updating his forecast with a reward prediction error. Through this process, the agent acquires information about value *but remains ignorant of probabilities*. This stands in sharp contrast with model-based reinforcement learning. There, in order to forecast future rewards, the agent forms a representation of how the world “behaves” and this can be done by learning the probabilities of all events given the current situation (i.e. “state transition probabilities”, [44]). A neural signature of probabilities but not value was observed during the learning period. In addition, model comparison showed that choices were better explained by a probability rather than a reinforcement learning algorithm. Thus both behavioral and biological data favored model-based over value-based reinforcement learning in our task. By showing both positive and negative BOLD response to event probabilities, the present study add to the previous literature on model-based reinforcement learning [45].

The functionality of the regions encoding probabilities deserves further discussion. Based on prior literature and our own results, we would argue that activation correlating with rare stimuli in occipital cortex reflects the visual exploration of the bin, while that in parietal and middle frontal gyrus captures the attention triggered by surprising events. Activation in hippocampus enhances encoding of rare stimuli. In contrast, the positive correlation between probabilities and activation in angular gyrus and medial prefrontal cortex would reflect the reactivation and reinforcement of past events in memory.

BOLD activity increased in the occipital cortex for rare stimuli and this could be due to visual exploration. When a rare payoff was sampled, its probability of occurrence increased. This might incite participants to identify its associated color and re-evaluate its relative importance by looking at all the colors and payoffs in the periphery of the bin. Previous studies have shown increased activation in the occipital cortex for visually incongruent stimuli and this effect seems to generalize to rare events in our study [46]. Rare events were also related to activation in the superior parietal cortex and middle frontal gyrus and this could be explained by attentional processes. Indeed, these regions were more activated during the task compared to the resting period (task-positive network) and have been related to attention or the oddball effect [17], [47]. Activity in the hippocampus was observed when a new stimulus was displayed, and the effect was stronger when the stimulus was rare. Lesions to the middle temporal area and the hippocampus can lead to amnesia and the inability to retain new information [48]. The BOLD response in the hippocampus suggests that rare events benefit from a better encoding when they occur. This is consistent with behavioural studies showing that surprising stimuli are better memorized [49].

Activity in the default network has been found to increase during tasks of theory of mind, mind wandering, and memory [50], [51]. On the contrary, it has been found to decrease when participants pay attention to external stimuli (task-negative network). This was also the case in our task because participants had to pay attention to the sampled payoffs. While controlling for the effect of the task, activity increased in several regions of the task-positive network when a rare stimuli was displayed. On the contrary, a BOLD response in angular gyrus and medial prefrontal cortex increased for frequent stimuli. A possible explanation for this novel result is that frequent stimuli attract less attention and hence allow for more resting-state introspection, the role traditionally assigned to the default mode network. This switch would be consistent with optimal use of the limited amount of energy available in the brain [52]. However, the switch would have to take place within the time frame of display of our stimuli (1 [s]). If this interpretation is indeed true, our findings would amount to evidence for high-frequency switching between elemental states of the brain, namely, attention and rest. An alternative explanation is that activity in angular gyrus and medial prefrontal cortex reflects a distinct process, namely, evidence accumulation. This process can be modeled as we did, in terms of learning of probabilities. A drift-diffusion approach [53] could be used instead, though this approach is fundamentally the same.

A cognitive mechanism that could explain the positive correlation between stimulus probability and brain activity is memory [29]. Cognitive psychologists have developed models centered on memory to explain how people judge the likelihood of events [54]. In these models, each outcome is encoded as a trace in memory. An event will be considered as probable if many traces are retrieved from memory in response to a probe (the payoff in our task). Neuroimaging studies have confirmed the involvement of parietal and medial prefrontal cortex in memory: activity in these areas predicts the successful recognition of items [31], [55]. Furthermore, many studies have demonstrated involvement of the default mode network in memory tasks [56], [57]. As a consequence, the positive brain response to stimulus probabilities in the angular gyrus and medial prefrontal cortex might reflect the activation and reinforcement of memory traces in reaction to a probe. This hypothesis is compatible with an “attention to memory” model developed in neuroscience [32], [58]. In this model, activation in the inferior parietal cortex reflects the attention captured by information retrieved from memory. Still, because fMRI can only recover correlation, other approaches like TMS are needed to determine the causal role of angular gyrus and medial prefrontal cortex in the acquisition and retrieval of event probabilities.

In decision neuroscience, the posterior cingulate and ventro-medial prefrontal are often involved in the judgement of value [59], [60]. For instance the ventro-medial prefrontal cortex is more activated when participants see the image of food they like [61], [62]. A recent study has shown that the time spent watching an item increased the likelihood to choose it and this type of behavior was well formalized by drift-diffusion models [63]. Preferences depend on the sensory characteristics of goods, but they are also shaped by our past experience and memories [64]. The reactivation of memory traces could partially explain why a key structure to evaluate the value of goods, the ventro medial prefrontal cortex, belongs to the default mode network and not to a task-positive or saliency network like the striatum [65]. Accordingly, a recent study has shown that affective value and associative processing shared a common substrate in medial prefrontal cortex [66].

Our study sheds new light on decision making under uncertainty when uncertainty is described as opposed to experienced [1]. In a task where decisions were based on experience, BOLD response to uncertain stimuli increased linearly with their probabilities of occurrence. This was confirmed in behavior: choices exhibited no bias is assessment of probabilities, in contrast to decision making based on description of available gambles [67]. Our results therefore cast doubt on the generalizability of probability weighting in prospect theory to decision making from experience [68].

In addition to a better understanding of the neural foundation of probability learning, the present study brings new knowledge concerning the representation of various kind of uncertainties in the brain [69]. Previous studies have linked uncertainty to activity in the insula [70], [71], but also in the anterior cingulate cortex [72]. In the present study, we found that BOLD response in insula and dorsal anterior cingulate were related to different forms of uncertainty. Activity in the anterior cingulate correlated with choice entropy which reflected uncertainty in making a choice. The later interpretation matches previous studies reporting BOLD response in dorsal anterior cingulate when a conflict existed between several responses [73] (difficulty of choice).

Activity in the insula increased with the gamble expected value and this effect was more pronounced when the outcome entropy was high. Entropy corresponds to the notion of expected uncertainty discussed by A. Yu and P. Dayan [74]. It is a function of probabilities only and thus does not depend on the value associated with the stimuli. An agent separating probability from value would favor entropy over reward volatility to estimate risk. When the outcome is a single and uncertain payoff, its standard deviation and entropy covary. This might explain why previous studies have found activation related to payoff standard-deviation in the insula [71]. Another possibility is that the insula becomes sensitive to entropy when participants learn state probabilities as in the present study and rely less on summary statistics like payoff mean and variance [75].

The general view that emerges from our study is that the brain does not only react to rewarding or surprising events, but also to likely events. When people observed uncertain stimuli, the average activity in the default mode network decreased compared to a resting condition. Nevertheless, the functional connectivity in this network increased and stimulus probabilities were positively correlated with BOLD response in angular gyrus and medial prefrontal cortex. Thus activity in these two regions signalled the accumulation of evidence (confirmatory signal). Brain response to uncertain stimuli increased linearly in probability and there was no evidence of probability weighting in choices. Further research is needed to test if the brain response to likely events reflects an activation of memory traces (internal world) or a lack of attention to the environment (external world).

## Methods

### Participants

Twenty-five students from the Université de Lausanne and the Ecole Polytechnique Fédérale de Lausanne were enrolled in the study. One participant was removed from the analysis because of significant head movements. Another, because her decisions to buy the gamble were random. The analyzed sample included 23 participants (10 women, 13 men; median age = 22, min = 19, max = 30; all right handed). The study took place at the University Hospital of Lausanne and was approved by its institutional review board. At the end of the experiment, students received 1/10 of their net play money in real currency, in addition to a 10 Frs (Swiss francs) participation reward.

To explain the task, the investigator read the instructions aloud and students played with one demonstration bin. They completed the task in a 3 Tesla MRI scanner. During the functional image acquisition, participants watched the display through goggles and indicated their decision to buy or to pass the gamble by pressing the left or right button of a response box. Participants learned probabilities and made decisions on 9 different bins. After bin 3 and 6, a resting phase of 60 [s] was introduced.

### Task

Payoffs were determined by the colors of balls drawn from a bin. Bins contained balls of different colors, with same-colored balls yielding the same payoff. The composition of the bin was hidden therefore probabilities were unknown. The time line for one example bin is shown in Fig. 2a. During the first sampling stage, 10 to 14 balls were drawn from the bin one after another and the associated payoff was displayed for 1 [s] at the center of the screen. Balls were drawn with replacement. Only stimuli representing the payoffs were shown. Colors were hidden states. These states could be inferred from the colored balls displayed in the periphery of the bin.

This learning phase was followed by a decision phase. The participant had to decide whether to buy the gamble or not for a certain price. After each choice, an additional ball was drawn. If the participant bought the gamble, he earned the payoff written on the ball minus the price. Otherwise, the payoff was 0 and the play money remained unchanged. Four choices were made without knowing the outcome in advance (active condition) and two choices were made while knowing the outcome in advance (control condition). For each of the six choices, a different price was posted. Prices were drawn from a uniform distribution between the minimum and maximum payoffs. After each decision, a message indicating that the gamble was bought or passed was shown (but the payoff was not shown to limit learning in the decision period). The total net payoff of the current decision period was displayed after the six choices.

The learning and decision phases were repeated with the same bin after changing the color-payoff association. That is, color probabilities remained unchanged (same composition of the bin), but each color was associated with a new payoff. It was thus adaptive to learn probabilities based on the color of the past drawings. In Fig. 2, the color-payoff association in panel a is reproduced in the top insert of panel b. For instance, red was associated with 57 in the sampling stage and with 67 in the resampling stage. Bins contained balls of 2, 5, or 10 different colors. The probabilities used to generate the drawings are represented by the histograms in Fig. 2b. Because balls were drawn with replacement, these probabilities remained constant during the sampling and resampling stages.

Nine different bins were presented in the task. Importantly, colors were randomly assigned to probabilities at the beginning of each new bin. Payoffs were randomly assigned to colors at the beginning of each sampling stage. As a consequence, payoff probabilities are orthogonal to payoff magnitudes and expected payoff. Uncertainty at decision time increased with both the number of possible payoffs and the payoff standard-deviation. To disentangle their effects, these two factors were manipulated independently (Fig. S5). See section *Task* in Text S1 for more details.

### Choice modeling

#### Learning phase

During the learning phase, probabilities were updated following Bayes' rule. In the mathematical formulation of the models, 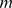 indexes the states 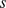 in the bin, 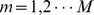, with 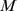 the number of states (2, 5 or 10 colors). So 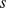 refers to a color (e.g., blue). The probability of state 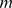 at drawing 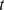 is a random variable 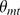, with 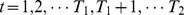 (with 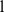 and 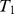 referring to the first and last drawing the initial sampling stage and 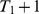 and 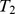 to the first and last drawing in the resampling stage). The vector of probabilities 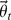 follows a Dirichlet distribution 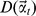. The PDF of 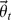 is given by:
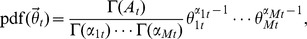
with 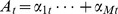. Let 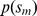 denote the true probability of state 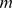. The point estimation 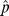 of this true probability is given by:
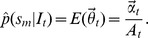
with 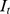 the information available at time 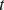.

We use 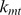 to indicate the number of times state 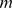 was observed at drawing 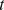. Before any drawing, 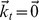. To specify the Dirichlet distribution at any time, we set 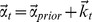. Without any knowledge of the composition of the bin, it is rational to assume that all colors have the same chance of occurrence when a new bin is encountered. Thus we chose 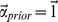. As a result and before any sample had been observed, 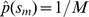 for all state 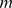. E.g., for a bin with 5 colors, all probabilities are expected to equal 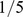. In model M4b (no probability learning), 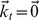 for all 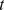, thus probabilities are not updated and remain equal to the priors. In model M4a (probabilities inferred from observations), 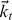 is set to 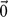 at the beginning of the sampling and resampling stages but it records the occurrence of states. This is equivalent to learning the probabilities without reference to colors. In model M4 (probabilities inferred from hidden states), 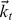 is set to 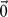 at the beginning of the initial sampling stage and it records the occurrence of states. It is not reset to 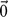 at the beginning of the resampling stage.

#### Decision phase

We first need to define a function 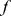 that associate a payoff 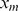 to each state 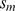. So this function links each color to a payoff. In the task, we only show the payoff in the center of the bin (stimulus), so the underlying state is inferred with the inverse function 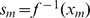. In model M2, the identity function was used to transform payoffs (net of the posted price 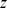). In models M4, M4a, M4b, and M5, a non-differentiable value function was used instead:
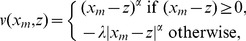
(1)with 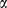 representing the diminishing sensitivity parameter and 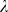 representing the loss aversion parameter.

In all models the identity function was used for probabilities, expect for model M5 which had a probability weighting function:
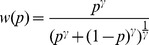
(2)where 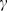 controls the S shape.

Subjective values and probabilities were multiplied and then summed over states to compute the expected value of the gamble:
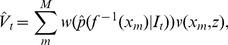
(3)To convert estimated values into choices, we used the softmax model. There, the probability that the gamble is bought is given by:
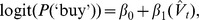
where 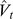 denotes the estimated value of the gamble at the end of the learning phase (i.e., at 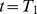 for the sampling stage, and 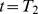 for the resampling stage). Nelder-Mead optimization was used to find the maximum likelihood (MLL) of this logistic regression. Search was repeated 5 times with different starting values. The best fit was retained (restarts yielded to very close results).

Outcome entropy at the end of the learning phase was computed with:
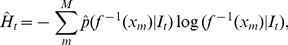
(4)with 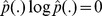 if 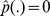. To compute the entropy of choices, state probabilities were replaced by choice probabilities derived from the softmax function.

## Supporting Information

Text S1 **Supplementary information.** Text S1 provides additional methods, results, figures, and tables.
(PDF)
