# Supplementary material for: Activity in Inferior Parietal and Medial Prefrontal Cortex Signals the Accumulation of Evidence in a Probability Learning Task
Source: PLoS Comput Biol. 2013 Jan 31;9(1):e1002895. doi: 10.1371/journal.pcbi.1002895 (PMC3561043; doi:10.1371/journal.pcbi.1002895)
Supplement: Text S1 — Supplementary information. Text S1 provides additional methods, results, figures, and tables. (PDF) [file pcbi.1002895.s001.pdf]

Activity in inferior parietal and medial prefrontal cortex signals the accumulation of evidence in a probability learning task.

Supplementary Information

Mathieu d'Acremont, Eleonora Fornari, and Peter Bossaerts

Contents

1 Behavioral choice **2**

2 Brain analysis **2**

2.1 Preprocessing 2

2.2 Voxel-based analysis 3

2.3 ROI analysis 3

2.4 Brain response to stimulus probabilities 4

2.5 Brain response to value and uncertainty 4

3 Task **4**

3.1 Instructions 4

3.2 Duration and message 5

4 Figures **6**

5 Tables **9**

1 Behavioral choice

Choices were recorded on 9 bins for 22 subjects. For the 23th subject, decisions were not recorded on the 8th bin because of a technical problem with the goggle. Analyses in the active condition were performed with 72 choices per subject (64 for the 23th subject). The total number of observations is reported in the footnote of each table.

Payoffs in the active decision condition were not revealed to the participants. They were revealed in the control decision condition. This information could be used to further learn probabilities during the decision period. However, they were not taken into account in the analyses because results showed that their inclusion did not lead to a better fit of the logistic regressions explaining choices. Thus probabilities were calculated based the drawings of the learning period only.

Due to the limited number of data, parameters of the prospect theory models (loss aversion, diminishing sensitivity, etc.) were estimated after merging the choices of all subjects and individual differences were ignored. However to test the prevalence of probabilistic sophistication, we compared the fit of models M4 (probabilities conditional on the hidden states) and M4a (probabilities conditional on the observed payoffs) at the individual level [1]. To avoid double dipping, a cross-validation was performed:

1. For each subject, prospect theory parameters were estimated based on all other subjects (hold-out) by maximizing the likelihood; this was done separately for models M4 and M4a;
2. For each subject, models M4 and M4a and the prospect theory parameters estimated in the previous step were used to predict their own choices, this led to a different likelihood for each subject; the BIC was then calculated based on the likelihood;
3. A paired t-test on the BIC was calculated to compare the fit of the two models; this way individual differences were taken into account; double dipping was avoided because of the hold-out (step 1).

The paired t-test showed that the BIC was significantly smaller for model M4 compared to M4a ($t(22) = -40.26, p < .001$). Thus the model based on hidden state inference better explained the observed choices. The BIC differences favored model M4 for all subjects (Fig. S2)

2 Brain analysis

BOLD fMRI acquisitions were performed with a 12 channels head coil on a 3 T Siemens Tim-Trio system. Functional MRI images were acquired with an EPI gradient echo T2*-weighted sequence (FA 90, TE 30, pixel size 3x3 [mm], acquisition time 1.9 [s], 32 slices of 3 [mm] thickness, covering the whole brain) with a TR = 2 [s].

High resolution morphological acquisition was acquired with a sagittal T1-weighted 3D gradient-echo sequence (MPRAGE), 160 slices (with voxel size of 1x1x1 [mm]), as a structural basis for brain segmentation and surface reconstruction.

Voxel-based and ROI analyses were done on 9 bins for 22 subjects. For the 23th subject, data were acquired for the first 7 bins only because of a technical problem with the goggle that occurred during the 8th bin. See table footnotes for the total number of observations.

2.1 Preprocessing

fMRI preprocessing steps, conducted with SPM8 (Wellcome Department of Cognitive Neurology, London, UK), included realignment of intra-session acquisitions to correct for head movement, normalization to a standard template (Montreal Neurological Institute template, MNI) to minimize inter-participant morphological variability and resampling to isotropic voxel of 2x2x2 [mm] to improve superposition of functional results and morphological acquisitions, and convolution with an isotropic Gaussian kernel (FWHM = 6 [mm]) to increase signal-to-noise ratio. The signal drift across acquisitions was removed with high-pass filter (only signals with a period < 240 [s] were included).

2.2 Voxel-based analysis

Subject was defined as a random factor in General Linear Models (GLM). The default orthogonalization of predictors was removed, otherwise the predictor order changes the results. The following GLM was set for the voxel-based analysis (GLM1). In the learning phase, the stimulus (payoff) was defined as an 1 [s] event in the GLM. The stimulus was modulated by its probability of occurrence calculated conditional on the past hidden states (model M4, Fig. S1). To model the absence of stimulus in the center and presence of colored ball in the periphery, a slice event was defined from the beginning of the learning phase to the end, but was "turned off" when the stimulus was displayed in the center of the bin.

A decision active event was defined starting from the display of the price ("Do you want to pay X Frs to play this bin") and the message asking for a response ("Yes / No"). This event lasted 3 [s]. In the control decision condition, a decision event was defined in the same way [1]. The decision active event was modulated by the Expected value, Outcome entropy, Expected value x Outcome entropy (interaction), and Choice entropy. The decision control event was modulated by the Net payoff (payoff minus price) and the Outcome entropy. Six events were defined for messages indicating: (1) the beginning of the sampling and resampling stages ("New composition / New outcomes" or "Same composition / New outcomes"), (2) the beginning of the learning phase ("Sampling period"), (3) the beginning of the decision phase ("Playing period"), (4) the call for a response ("Yes / No"), (5) the response feed-back ("Outcome recorded" or "Gamble passed"), and (6) the payoff feedback ("our net payoff [...]"). The payoff feed-back message was modulated by the total net payoff of the current decision phase (sum of the six payoffs minus sum of the six prices).

To test the effect of value, the stimulus was modulated by a prediction error (GLM2). The prediction error was the expected payoff calculated after the drawing was revealed minus the expected payoff calculated before the drawing was revealed (change in expected payoff). Expected payoff was calculated with probabilities inferred from states (model M4). To analyze the effect of valence, separate events were defined within a single GLM based on the sign of the prediction error they generated (GLM3).

2.3 ROI analysis

To define ROIs, GLM1 was modified to include two types of probabilities in the learning phase (GLM4): (a) the probabilities calculated conditional on the hidden states (model M4, Fig. S1) and (b) the probabilities calculated conditional on the observed payoffs (model M4a, Fig. S1). Secondly, a contrast assessing the joint effect of the two types of probabilities was defined. With this approach, none of the two covariates was favored in the definition of the ROIs. Significant voxels found at the group level were used to define 3 ROIs encoding probabilities: medial prefrontal cortex, left and right angular gyrus. Significant voxels were used to define 2 ROIs encoding entropy during the active decision phase: the bilateral insula and the dorsal anterior cingulate. To avoid circularity or "double dipping", ROIs for each individual were determined based on the data of all other participants [2].

The GLM for the ROI analysis was the same as the other GLMs except that a different event was defined for each stimulus (display of a payoff) and each decision message ("Do you want to pay X Frs to play this bin"). These events were not modulated by covariates (GLM5). This GLM was fitted to the brain functional data and Marsbar toolbox was used to extract the first component score of all voxels in a given ROI [3]. This was done for each subject separately. Because ROIs for each subject were estimated without the subject himself, circularity was avoided. Estimated betas were imported in R [4]. In R, mixed linear and mixed non-linear models were computed to predict these GLM betas estimated with Marsbar (an individual beta was obtained for each stimulus and each decision). In line with the GLMs, Subject was defined as a random factor in R mixed models. Probabilities were centered at 0.5 (except in Tables S8 & S9), the number of colors was centered at 5, and all other predictors were scaled. The total number of observations is reported in the table footnotes.

Functional connectivity was analyzed with Psycho-Physiological Interaction (PPI) in SPM. ROIs encoding probabilities were used as seed regions. A first analysis was conducted on all acquired images and the learning phase was defined as an experimental factor (GLM6). This factor equaled 1 during the learning phase, 0 otherwise. The interaction between this factor and BOLD response was entered in the PPI to test if connectivity increased during the learning phase. The GLM also included a predictor for the 1 [s] stimuli shown during the learning period and its probability. Thus the PPI interaction

[1] Defining decision events starting from the display of the price and ending with the participant response led to the same results

term tested for an increase in connectivity during the learning period after controlling for the stimulus presentation and its probability of occurrence. A second connectivity analysis was conducted on the images acquired during the resting phase only and did not include an experimental factor (GLM7). Like for all other voxel-based GLMs, connectivity analyses included realignment regressors to control for head motion [5].

2.4 Brain response to stimulus probabilities

We conducted supplementary analyses to test if the brain was encoding the probabilities of the stimuli conditional on the hidden states or the observed payoffs. The two type of probabilities converge as the number of sample increases and are thus strongly related. They are identical in the initial sampling phase ($r = 1$), and highly correlated in the resample phase ($r = .92, p < .001$, see Fig. S1). As a consequence, when stimulus probabilities inferred from past payoffs instead of past colors were entered in the GLM, similar activation was observed. It is thus difficult to disentangle their effect at the level of voxels.

To increase statistical power, analysis on the ROIs encoding probabilities was performed. The ROIs are the clusters of significant voxels found with GLM4 and the contrast summing the effect the two types of probabilities (inferred from the hidden states or the observed payoffs). Average BOLD response in ROIs during the resampling stage (GLM5) was used as a criterion in R mixed-linear regressions (the two types of probabilities do not differ in the first sampling stage). Results showed a significant effect of the probabilities inferred from the hidden states ($P = .02$) but not for the probabilities inferred from the observed payoffs ($P = .33$, Table S20). The location of the ROI did not interact with probabilities inferred from the hidden states ($F(2,7340) = 0.73$, $P = .48$) or the observed payoffs ($F(2,7340) = 0.63$, $P = .53$). Thus, the three ROIs appeared to encode probabilities inferred from hidden states and not the observed consequences.

2.5 Brain response to value and uncertainty

In the decision period, voxel-based analysis showed that BOLD response in dorsal anterior cingulate cortex increased with choice entropy. BOLD response in bilateral insula increased for gambles combining high expected value and high outcome entropy. Voxels significantly activated by these variables were used to define ROIs (GLM4). Average BOLD response in ROIs during the decision stage (GLM5) was used as a criterion in R mixed-linear regressions. A location variable was created and set to 1 for the anterior cingulate and 0 for the left and right insula. Results showed a significant and positive interaction Location x Choice entropy ($t(4894) = 4.03, p < .001$). Thus the effect of choice entropy was significantly stronger in the dorsal anterior cingulate. The triple interaction Location x Expected value x Outcome entropy was not significant ($t(4890) = -0.60, p = .55$). Thus the interaction Expected value x Outcome entropy was not stronger in the insula. Overall, ROI analysis confirmed that the dorsal anterior cingulate cortex encoded choice entropy. A specific role of the insula in encoding outcome entropy was found with voxel-based analysis but could not be confirmed on ROIs. Further studies are thus needed to test the existence of a complete double dissociation.

3 Task

3.1 Instructions

In the experiment, you will be given the opportunity to buy gambles at prices we post. You will start out with play money. If you decide to buy a gamble, the price we quoted is subtracted from your play money, while the earnings from the gamble is added. The outcome of each gamble is based on a random drawing of a ball from a bin. Balls within each bin are distinguished by color. There may be many balls of the same color, but you will not know how many... To give you an idea, the computer will draw several times a ball from the bin.

Like in standard lotteries, each ball is labeled with a number. This number is the same for balls of the same color. This number determines how much you earn when a ball is drawn.... For instance, if the orange balls are labeled “9”, you make 9 francs every time an orange ball is drawn.

Occasionally, we may change the labels without changing the composition of the bin (number of balls of each color). We will tell you when that happens... At that point, the computer will draw several times one ball, before you make any

decision whether to buy into the gamble or not.

Whenever we draw a ball from a bin, we replace it, so the number of balls does not change from one drawing to another, until of course we change the composition of the bin.

Before you decide to buy into a gamble, we will tell you whether the outcome will be known in advance. If it is not the instructions are in red. When we tell you the outcome in advance the instructions are in green. In any case, a "yes/no" instruction will appear after a few seconds. Then you have 15 seconds to decide to buy into a gamble. A countdown displays how much time you have left. If you don't decide within 15 sec, the computer will make the decision at random.

So, the sequence of events is as follows. First, we show you a bin: you will be told which colors are in the bin and what label (payoff) is associated with each color, but not how many balls of each color there are. Then the computer will draw several times a ball from the bin and you'll see the corresponding outcome (payoff). Then we move to the playing period: we quote a price and ask you whether you want to buy the gamble. If you don't (push "right") then the gamble is passed; if you do (push "left"), then the price and the outcome are recorded. Six prices will be presented successively and each time you have to decide whether or not to buy the gamble. You will know the outcome in advance for playing rounds 3 and 4 (instructions in green). You will not in the other playing rounds (1, 2, 5 and 6; instructions in red). At the end of the playing rounds, the cumulative net payoff will be displayed. That net payoff will be added to your play money. After the playing period, we either change the labels of the balls keeping the composition of the bin the same, or we change both. Don't worry - we will tell you which case applies.

The money you earn (play money plus earnings from gambles you buy minus the prices you paid) is yours to keep, in addition to the traditional sign-up reward.

3.2 Duration and message

The task started with a message displaying the initial play money. Each bin was preceded by a message indicating the beginning of the initial sampling stage (8 [s]), followed by a message indicating the beginning of the learning phase (4 [s]). The first drawing was shown 1.5 [s] after the disappearance of the last message. During the learning phase, each outcome was displayed during 1 [s]. The SOA between drawings followed a uniform distribution and ranged between 4 and 5 [s] (jittering).

After the end of the learning phase, a message indicated the beginning of the decision phase (4 [s]). The message showing the price in the decision phase "Do you want to pay X Frs to play this bin" lasted 3 [s]. After these 3 [s], the message "Yes / No" was displayed below the price message. The participant had to respond before 15 [s], otherwise the decision to buy the gamble or not was taken randomly by the computer. A count-down indicated the time left (updated every 5 [s]). After the participant answered, a feedback message indicated that the "Outcome was recorded" if the gamble was bought, or that the "Gamble was passed" otherwise (1 [s]). The gamble was played with 6 different prices: 2 in the active phase, 2 in the control phase, and 2 again in the active phase. After the 6 rounds, a message displayed the total net payoff of the current sampling stage along with the updated play money (10 [s]).

Then a message indicated the beginning of the resampling stage (8 [s]) followed by a message indicating the beginning of the learning phase (4 [s]). The first drawing was shown 1.5 [s] after the disappearance of the last message.

After bin 3 and 6, a resting period of 60 [s] was introduced. The message "Resting time (60 sec)" was displayed in the center of the screen.

4 Figures

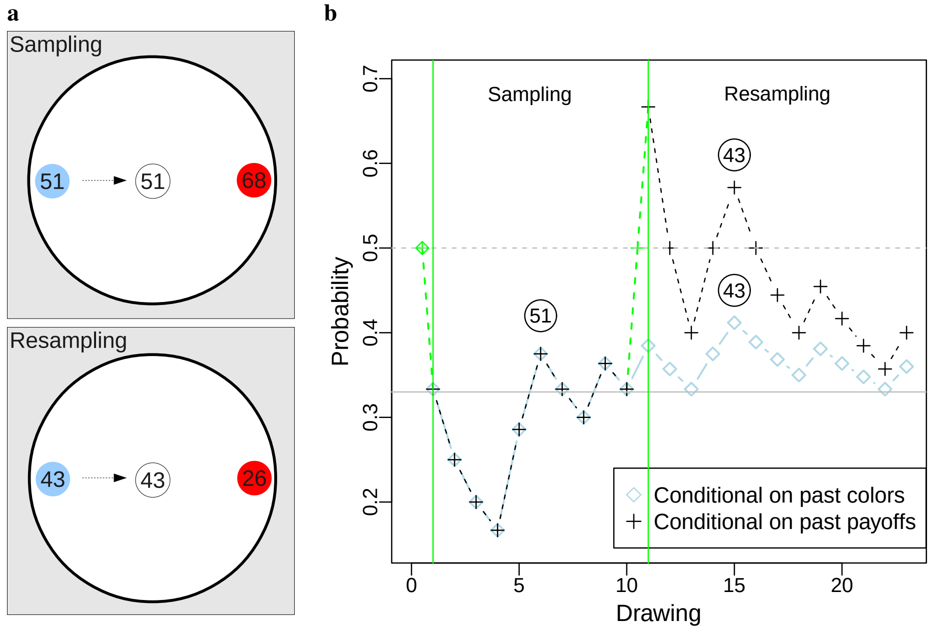

Figure S1: Type of inference. (a) Example of a two-color bin. The true probabilities equal 1/3 for blue and 2/3 for red. The payoff associated with each color change in the resampling stage but not the probabilities of the colors. **(b)** The figure displays the evolution of the posterior probabilities of the payoff associated to the blue ball for a given sequence of drawings. The updating process starts with a probability equal to 0.5 (horizontal dashed gray line). The first vertical green line indicates the first drawing of the initial sampling stage. The second vertical line indicates the first drawing after a change in the color-payoff association, keeping color probabilities the same (resampling stage). The dashed black line shows the probability calculated conditional on the observed payoffs (model M4a). It ignores that the same colors generate the payoffs in the sampling and resampling stages. Therefore, the probability is reset to 1/2, prior to the first drawing of the resampling stage. The dashed blue line shows the probability calculated conditional on the hidden states (model M4). The probability is not reset to the prior at the beginning of the resampling stage and thus converges faster to the true probability (horizontal gray line). The probability regressed on BOLD response is the probability of the payoff shown in the center of the screen during the learning phase (stimulus). For instance, in the resampling stage it is the probability of 43 if a blue ball is drawn and of 26 if a red ball is drawn. The probability of the same stimulus (e.g. “43”) can be inferred from the history of the hidden states (model M4) or the history of observed payoffs (model M4a).

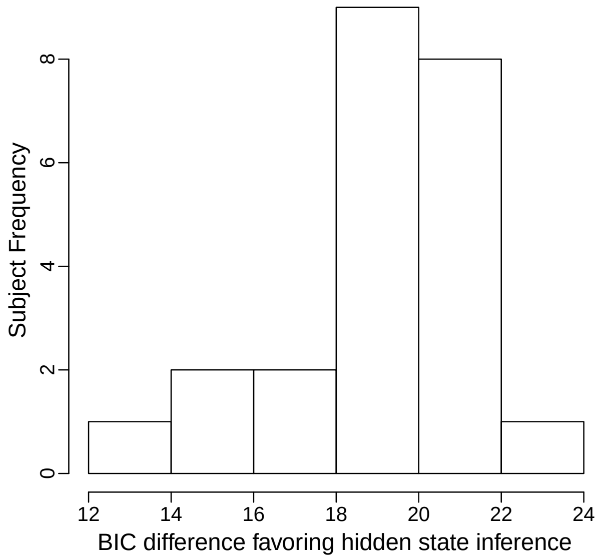

Figure S2: Individual differences. Gamble expected values inferred from hidden states (model M4) and observed payoffs (model M4a) were used to explain choices. The difference between the two BIC (M4a-M4) was calculated for each individual. It can be seen in the histogram that all differences were positive. Thus all participants were learning probabilities based on the state history.

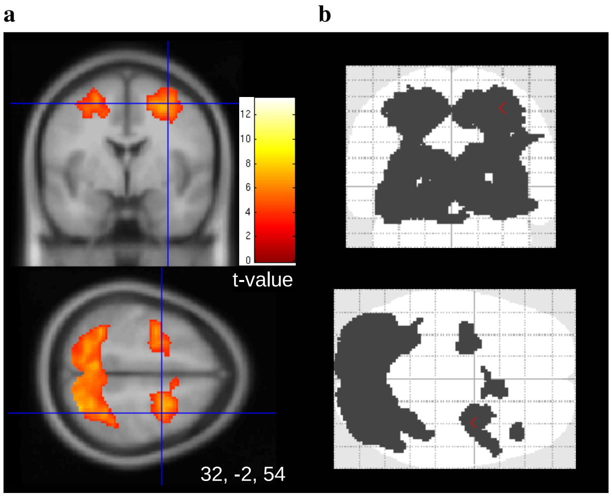

Figure S3: Brain response to improbable stimuli. (a) When a rare stimulus was observed in the learning period, BOLD activity increased in the occipital, superior parietal, and middle frontal gyrus increased. A significant effect was also observed in bilateral hippocampus (see Supplementary Tables). **(b)** Overlap between voxels encoding stimulus improbabilities (panel a) and the task-positive network (Fig. 6b, red voxels).

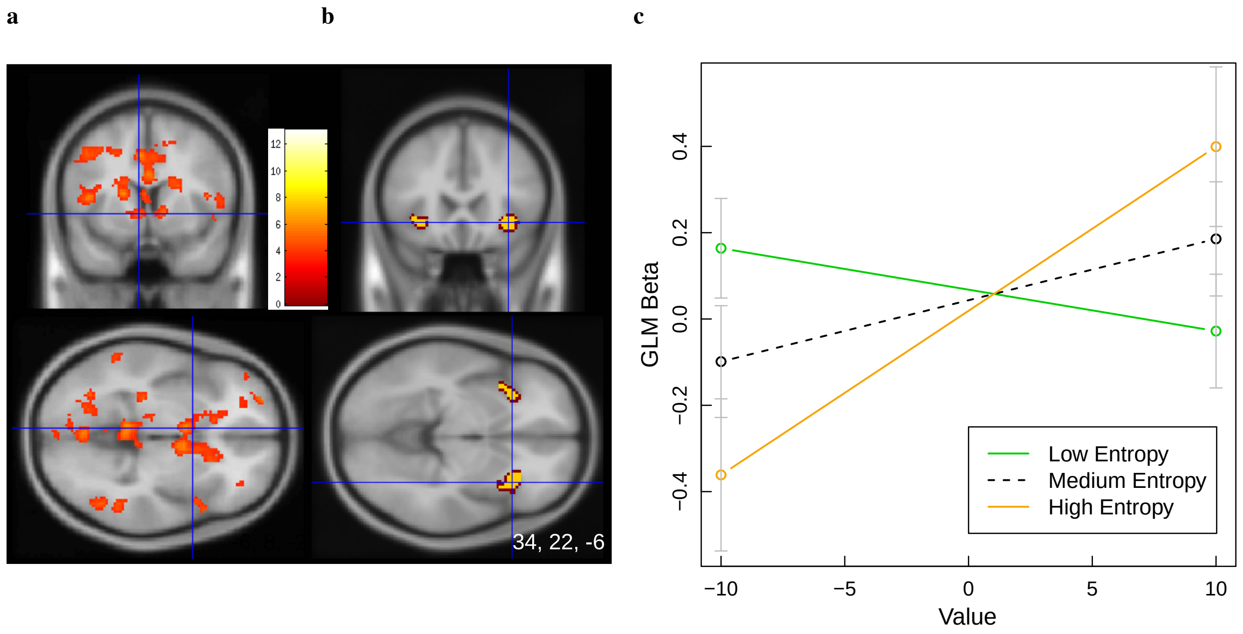

Figure S4: Value and entropy. **(a)** BOLD response to gamble expected value (net of the price) at decision time. Activation was mainly observed in the caudate but reached other regions of the brain. **(b)** Cross validation used to define the insula ROI. To avoid circularity, ROIs for each individual were determined based on the data of all other participants. ROI voxels common to all participants are in yellow (AND operator). ROI voxels belonging to at least one participant are in red (OR operator). This representation shows to which extent ROI definitions varied in the cross validation. **(c)** Overall, activity in bilateral insula increased with gamble expected value. The effect of value was amplified when the outcome was uncertain due to a high entropy (predicted activation and standard error).

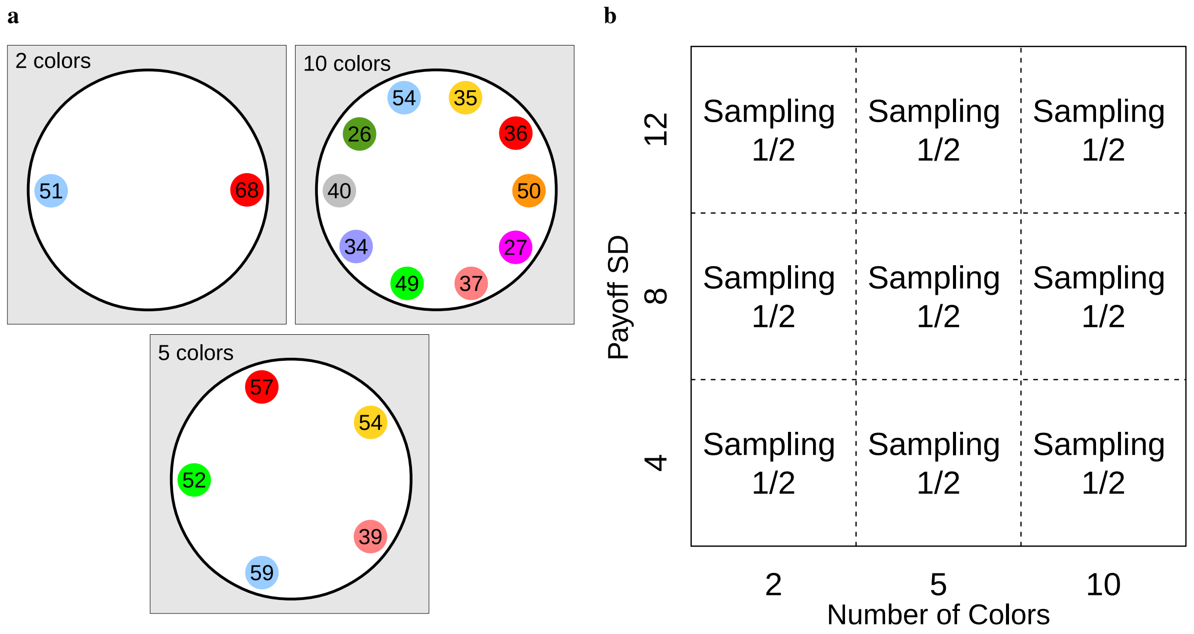

Figure S5: Factorial design. **(a)** Example of bins with 2, 5, or 10 colors. **(b)** Payoff standard deviation (SD 4, 8 or 12) was manipulated independently of the number of colors in the bin. This yielded to a total of 9 bins. Payoffs in the resampling stage were chosen so that the expected payoff changed, but not the payoff standard deviation.

5 Tables

Table S1: Voxel-Based Analysis: BOLD Response Regressed on Stimulus minus Absence of Stimulus

Cluster	p_{FDR}	k_E	p_{unc}							
Local Max				p_{FDR}	t	z	p_{unc}	x	y	z
Occipital/Hippocampus	0.000	26677	0.000							
L Cuneus				0.000	16.21	7.44	0.000	−12	−88	26
L Calcarine				0.000	14.33	7.10	0.000	−2	−90	14
R Calcarine				0.000	14.27	7.09	0.000	16	−64	10
R Mid+Sup Frontal Gyrus	0.000	685	0.000							
R Sup Frontal				0.001	9.14	5.81	0.000	26	6	64
R Mid Frontal				0.026	6.22	4.68	0.000	30	−2	58
R Mid Frontal				0.070	5.56	4.35	0.000	22	12	48
R Mid Frontal Gyrus	0.000	613	0.000							
R Mid Frontal				0.007	7.05	5.05	0.000	38	52	8
R Mid Frontal				0.131	5.13	4.12	0.000	48	44	16
R Mid Frontal				0.132	5.12	4.11	0.000	52	28	32
L Mid+Sup Frontal Gyrus	0.000	461	0.000							
L Sup Frontal				0.017	6.51	4.81	0.000	−26	2	68
L Sup Frontal				0.028	6.15	4.64	0.000	−26	−6	60
L Mid Frontal				0.106	5.29	4.20	0.000	−26	2	54

Height threshold: T = 3.50, p = 0.001; Extent threshold: k = 300 voxels, p = 0.000.

Table S2: Voxel-Based Analysis: BOLD Response Regressed on Stimulus Probabilities

Cluster	p_{FDR}	k_E	p_{unc}							
Local Max				p_{FDR}	t	z	p_{unc}	x	y	z
R Angular Gyrus	0.000	457	0.000							
R Angular				0.002	9.68	5.99	0.000	56	−60	38
R Angular				0.010	7.49	5.23	0.000	50	−66	42
R Angular				0.023	6.92	4.99	0.000	58	−62	28
Medial Prefrontal Cortex	0.000	1864	0.000							
Ventral Ant Cingulate				0.005	8.51	5.60	0.000	−4	24	−6
Sup Frontal Gyrus				0.136	5.52	4.33	0.000	−6	46	52
Ant Cingulate				0.178	5.19	4.15	0.000	0	44	10
L Angular Gyrus	0.000	504	0.000							
L Angular				0.005	8.26	5.52	0.000	−52	−70	30
L Angular				0.005	8.14	5.47	0.000	−56	−62	36
L Angular				0.007	7.76	5.33	0.000	−46	−72	40

Height threshold: T = 3.50, p = 0.001; Extent threshold: k = 100 voxels, p = 0.016.

Table S3: Voxel-Based Analysis: BOLD Response Regressed on Stimulus Improbabilities

Cluster / Local Max	p_{FDR}	k_E	p_{unc}	p_{FDR}	t	z	p_{unc}	x	y	z
Occipital	0.000	21784	0.000							
L Fusiform				0.000	13.37	6.91	0.000	−34	−76	−14
R Occipital Sup				0.000	13.23	6.88	0.000	26	−66	32
R Inf Occipital				0.000	11.00	6.36	0.000	42	−70	−14
R Frontal	0.000	1222	0.000							
R Sup Frontal Gyrus				0.004	7.14	5.08	0.000	26	4	54
R Suppl Motor				0.071	5.17	4.14	0.000	6	14	48
R Mid Cingulate				0.111	4.90	3.99	0.000	12	18	42
L Frontal	0.000	690	0.000							
L Precentral				0.004	7.03	5.04	0.000	−34	−6	52
L Sup Frontal				0.017	6.10	4.62	0.000	−24	−4	56
L Sup Frontal				0.071	5.17	4.14	0.000	−18	−6	50
L Hippocampus	0.005	175	0.002							
L Hippocampus				0.012	6.32	4.72	0.000	−20	−30	−4
L Hippocampus				0.153	4.72	3.88	0.000	−20	−30	8
R Precentral	0.002	226	0.001							
R Precentral				0.021	5.96	4.55	0.000	46	6	32
R Hippocampus	0.001	306	0.000							
R Hippocampus				0.023	5.89	4.51	0.000	22	−30	2
R Hippocampus				0.458	4.06	3.47	0.000	26	−22	−6
R Thalamus				0.841	3.62	3.17	0.001	12	−20	12
R Mid Frontal Gyrus	0.010	144	0.005							
R Mid Frontal Gyrus				0.029	5.73	4.43	0.000	40	32	22

Height threshold: T = 3.50, p = 0.001; Extent threshold: k = 100 voxels, p = 0.016.

Table S4: Voxel-Based Analysis: BOLD Response Regressed on Probabilities / Positive Stimuli

Cluster / Local Max	p_{FDR}	k_E	p_{unc}	p_{FDR}	t	z	p_{unc}	x	y	z
Medial Prefrontal Cortex	0.000	1368	0.000							
Ventral Ant Cingulate				0.008	8.71	5.67	0.000	−4	24	−2
Ant Cingulate				0.053	6.53	4.82	0.000	0	52	12
Ant Cingulate				0.089	5.84	4.49	0.000	−4	52	2
R Angular Gyrus	0.001	286	0.000							
R Angular				0.051	6.87	4.97	0.000	56	−64	34
R Angular				0.062	6.38	4.75	0.000	56	−66	26
R Angular				0.083	5.91	4.52	0.000	66	−42	36
L Angular	0.002	253	0.000							
L Angular				0.051	6.72	4.90	0.000	−46	−72	40
L Angular				0.051	6.65	4.87	0.000	−54	−64	38
L Angular				0.071	6.13	4.63	0.000	−52	−70	32
Sup Frontal Gyrus	0.000	400	0.000							
L Sup Frontal Gyrus				0.051	6.69	4.89	0.000	−10	48	46
R Sup Frontal Gyrus				0.071	6.14	4.64	0.000	12	36	48
L Sup Frontal Gyrus				0.101	5.70	4.42	0.000	−8	42	54

Height threshold: T = 3.50, p = 0.001; Extent threshold: k = 100 voxels, p = 0.012.

Table S5: Voxel-Based Analysis: BOLD Response Regressed on Probabilities / Negative Stimuli

Cluster / Local Max	p_{FDR}	k_E	p_{unc}	p_{FDR}	t	z	p_{unc}	x	y	z
R Angular Gyrus	0.000	378	0.000							
R Angular				0.022	7.77	5.33	0.000	56	−60	40
R Angular				0.022	7.46	5.21	0.000	50	−66	42
R Angular				0.084	5.95	4.55	0.000	56	−64	30
L Angular Gyrus	0.000	451	0.000							
L Angular				0.022	7.24	5.13	0.000	−52	−70	30
L Angular				0.056	6.33	4.73	0.000	−46	−62	48
L Angular				0.369	4.78	3.92	0.000	−42	−78	34
Medial Prefrontal Cortex	0.000	358	0.000							
Ventral Ant Cingulate				0.023	7.05	5.04	0.000	−4	26	−8
VMPFC				0.366	4.86	3.96	0.000	0	42	−12
VMPFC				0.895	3.78	3.28	0.001	0	52	−6

Height threshold: T = 3.50, p = 0.001; Extent threshold: k = 100 voxels, p = 0.013.

Table S6: ROI Analysis: Effect of Locations and Nbr. of States

Variable	*Estimate*	*Lower*	*Upper*	*SE*	*Df*	*t*	*p*
Fixed effect							
Medial Prefrontal	−0.111	−0.592	0.371	0.246	14670	−0.45	0.652
L Angular	0.257	−0.219	0.734	0.243	14670	1.06	0.290
R Angular	0.593*	0.130	1.057	0.236	14670	2.51	0.012
Probability	2.086***	1.221	2.951	0.441	14670	4.73	0.000
Nbr States	0.028	−0.049	0.106	0.040	14670	0.71	0.476
Prob x L Angular	0.186	−0.527	0.899	0.364	14670	0.51	0.610
Prob x R Angular	−0.066	−0.733	0.600	0.340	14670	−0.19	0.846
Prob x Nbr States	0.045	−0.184	0.275	0.117	14670	0.39	0.698
Random effect (SD)							
Medial Prefrontal	1.076	–	–	–	–	–	–
L Angular	1.090	–	–	–	–	–	–
R Angular	1.055	–	–	–	–	–	–
Probability	1.120	–	–	–	–	–	–
Nbr States	0.108	–	–	–	–	–	–
Prob x L Angular	0.807	–	–	–	–	–	–
Prob x R Angular	0.522	–	–	–	–	–	–
Prob x Nbr States	0.382	–	–	–	–	–	–
Error	3.390	–	–	–	–	–	–

*0 not included in the 95% Confidence Interval; Nbr. data = 14700;
The F statistic for the interaction ROI Location x Probability was not significant, F(2, 14670) = 0.23, p = .79.

Table S7: ROI Analysis: BOLD Response Regressed on Probabilities for each Nbr. of States

Variable	*Estimate*	*Lower*	*Upper*	*SE*	*Df*	*t*	*p*
Fixed effect							
Medial Prefrontal	−0.132	−0.706	0.441	0.293	14670	−0.45	0.651
L Angular	0.228	−0.247	0.704	0.243	14670	0.94	0.347
R Angular	0.604**	0.145	1.062	0.234	14670	2.58	0.010
2 States	−0.017	−0.433	0.398	0.212	14670	−0.08	0.935
10 States	0.301	−0.427	1.029	0.371	14670	0.81	0.417
Prob:2 States	1.788***	1.037	2.539	0.383	14670	4.67	0.000
Prob:5 States	2.158**	0.757	3.560	0.715	14670	3.02	0.003
Prob:10 States	2.703**	0.778	4.627	0.982	14670	2.75	0.006
Random effect (SD)							
Medial Prefrontal	1.229	–	–	–	–	–	–
L Angular	1.116	–	–	–	–	–	–
R Angular	1.072	–	–	–	–	–	–
2 States	0.736	–	–	–	–	–	–
10 States	0.539	–	–	–	–	–	–
Prob:2 States	1.085	–	–	–	–	–	–
Prob:5 States	2.163	–	–	–	–	–	–
Prob:10 States	1.406	–	–	–	–	–	–
Error	3.380	–	–	–	–	–	–

*0 not included in the 95% Confidence Interval; Nbr. data = 14700.

Table S8: ROI Analysis: BOLD Response Regressed on Stimulus Probabilities (linear)

Variable	*Estimate*	*Lower*	*Upper*	*SE*	*Df*	*t*	*p*
Fixed effect							
β_0	$-0.810{*}{*}{*}$	-1.273	-0.348	0.236	14676	-3.44	0.001
β_1	$1.844{*}{*}{*}$	1.289	2.399	0.283	14676	6.51	0.000
Random effect (SD)							
β_0	1.101	–	–	–	–	–	–
β_1	1.192	–	–	–	–	–	–
Error	3.472	–	–	–	–	–	–

*0 not included in the 95% Confidence Interval; Nbr. data = 14700;
β_0 = intercept, β_1 = slope.

Table S9: ROI Analysis: BOLD Response Regressed on Stimulus Probabilities (non-linear)

Variable	*Estimate*	*Lower*	*Upper*	*SE*	*Df*	*t*	*p*
Fixed Effect							
β_0	$-0.63{*}{*}$	-1.05	-0.20	0.22	14675	-2.91	.00
β_1	$1.63{*}{*}{*}$	1.39	1.86	0.12	14675	13.38	.00
γ'	0.49	-0.29	1.26	0.39	14675	1.23	.22
Random Effect							
β_0	0.90	–	–	–	–	–	–
β_1	0.00	–	–	–	–	–	–
ε	3.48	–	–	–	–	–	–

*0 not in 95% Confidence Interval; Nbr. data = 14700; β_0 = intercept, β_1 = slope, $\gamma' = \gamma - 1$, γ = probability weighting, ε = error;
The random effect for γ was negligeable and was thus removed from the model.

Table S10: Voxel-Based Analysis: Increase in Connectivity during the Learning Phase

Cluster / Local Max	p_{FDR}	k_E	p_{unc}	p_{FDR}	t	z	p_{unc}	x	y	z
Precuneus/Cingulate	0.000	5185	0.000							
Mid Cingulate				0.000	14.58	7.15	0.000	0	−18	34
Post Cingulate				0.000	12.28	6.67	0.000	4	−46	20
Post Cingulate				0.000	10.98	6.35	0.000	−4	−48	24
L Angular Gyrus	0.000	1169	0.000							
L Angular				0.000	11.24	6.42	0.000	−54	−62	32
Medial Prefrontal Cortex	0.000	3846	0.000							
Sup Frontal				0.000	10.90	6.33	0.000	−14	48	38
Sup Frontal				0.001	9.23	5.84	0.000	−6	38	32
Sup Frontal				0.001	8.80	5.70	0.000	10	44	44
Occipital	0.000	1283	0.000							
R Calcarine				0.054	5.85	4.49	0.000	14	−90	12
L Lingual				0.072	5.66	4.40	0.000	−8	−96	−16
R Fusiform				0.074	5.62	4.38	0.000	28	−62	−8
R Angular Gyrus	0.000	391	0.000							
R Angular				0.124	5.14	4.12	0.000	62	−50	40
R Angular				0.129	5.11	4.10	0.000	48	−54	34
R Angular				0.132	5.07	4.08	0.000	58	−62	30

Height threshold: T = 3.50, p = 0.001; Extent threshold: k = 300 voxels, p = 0.000.

Table S11: Voxel-Based Analysis: BOLD Response Regressed on Active minus Control Decision

Cluster / Local Max	p_{FDR}	k_E	p_{unc}	p_{FDR}	t	z	p_{unc}	x	y	z
Occipital/Hippocampus/Striatum	0.000	20858	0.000							
R Cuneus				0.001	10.63	6.26	0.000	8	−86	28
L Cuneus				0.001	10.50	6.22	0.000	−2	−84	20
R Sup Occipital				0.001	10.08	6.10	0.000	20	−82	38
R Insula/Inf Frontal	0.000	1599	0.000							
R Insula				0.004	7.47	5.22	0.000	38	24	−8
R Inf Frontal				0.018	6.44	4.78	0.000	54	10	20
R Insula				0.031	6.04	4.59	0.000	30	32	−4

Height threshold: T = 3.50, p = 0.001; Extent threshold: k = 300 voxels, p = 0.000.

Table S12: Voxel-Based Analysis: BOLD Response Regressed on Expected Value

Cluster<br>Local Max	p_{FDR}	k_E	p_{unc}	p_{FDR}	t	z	p_{unc}	x	y	z
Undefined	0.000	24136	0.000							
R Caudate				0.090	8.12	5.47	0.000	26	24	20
L Supramarginal				0.090	7.98	5.41	0.000	−44	−42	34
Cingulate Gyrus				0.090	7.80	5.35	0.000	−14	−38	36
L Frontal	0.008	169	0.000							
L Sup Frontal Gyrus				0.143	5.76	4.45	0.000	−28	60	0
Mid Frontal Gyrus				0.537	4.07	3.48	0.000	−38	50	0
Undefined	0.005	195	0.000							
R Operculum				0.171	5.44	4.29	0.000	44	8	12
R Inf Frontal Gyrus				0.198	5.22	4.17	0.000	54	14	4
R Insula				0.386	4.44	3.71	0.000	48	10	−2
R Orbito frontal	0.018	136	0.001							
R Orbito Frontal				0.198	5.24	4.18	0.000	28	42	−14
R Orbito Frontal				0.286	4.73	3.89	0.000	38	50	−12
R Orbito Frontal				0.507	4.14	3.52	0.000	34	42	−4
R Inf Frontal Gyrus	0.005	189	0.000							
R Inf Frontal Gyrus				0.212	5.14	4.12	0.000	50	18	22
R Inf Frontal Gyrus				0.420	4.35	3.65	0.000	42	22	16
R Inf Frontal Gyrus				0.507	4.14	3.52	0.000	58	18	30

Height threshold: T = 3.50, p = 0.001; Extent threshold: k = 100 voxels, p = 0.003.

Table S13: Voxel-Based Analysis: BOLD Response Regressed on Expected Value x Outcome Entropy

Cluster<br>Local Max	p_{FDR}	k_E	p_{unc}	p_{FDR}	t	z	p_{unc}	x	y	z
R Insula	0.011	165	0.000							
R Insula				0.257	6.42	4.77	0.000	32	24	−6
L Insula	0.022	124	0.001							
L Insula				0.721	5.11	4.10	0.000	−34	14	−14
L Insula				0.742	4.85	3.96	0.000	−30	24	−8
L Insula				0.742	4.72	3.88	0.000	−36	16	−2

Height threshold: T = 3.50, p = 0.001; Extent threshold: k = 100 voxels, p = 0.003.

Table S14: ROI Analysis: BOLD Response Regressed on Expected Value x Outcome Entropy

Variable	*Estimate*	*Lower*	*Upper*	*SE*	*Df*	*t*	*p*
Fixed effect							
L Insula	−0.083	−0.287	0.121	0.104	3253	−0.80	0.423
R Insula	0.220∗∗	0.071	0.369	0.076	3253	2.90	0.004
Expected Value	0.065∗	0.012	0.118	0.027	3253	2.39	0.017
Entropy	−0.038	−0.118	0.042	0.041	3253	−0.94	0.348
Entropy x EV	0.078∗∗∗	0.035	0.122	0.022	3253	3.51	0.000
Random effect (SD)							
L Insula	0.484	–	–	–	–	–	–
R Insula	0.323	–	–	–	–	–	–
Expected Value	0.097	–	–	–	–	–	–
Entropy	0.175	–	–	–	–	–	–
Entropy x EV	0.067	–	–	–	–	–	–
Error	1.001	–	–	–	–	–	–

*0 not included in the 95% Confidence Interval; Nbr. data = 3280.

Table S15: Voxel-Based Analysis: BOLD Response Regressed on Choice Entropy

Cluster	p_{FDR}	k_E	p_{unc}							
Local Max				p_{FDR}	t	z	p_{unc}	x	y	z
L Occipital/Parietal	0.000	385	0.000							
L Sup Occipital				0.205	5.94	4.54	0.000	−20	−70	48
L Sup Parietal				0.221	5.52	4.33	0.000	−24	−80	44
L Mid Occipital				0.436	4.81	3.93	0.000	−28	−74	32
R Occipital/Parietal	0.000	404	0.000							
R Sup Occipital				0.205	5.90	4.52	0.000	30	−74	46
R Sup Parietal				0.367	5.10	4.10	0.000	14	−70	58
R Precuneus				0.457	4.60	3.81	0.000	16	−60	42
Ant Cingulate/Sup Frontal	0.000	395	0.000							
Sup Frontal Gyrus				0.205	5.82	4.48	0.000	−4	24	40
Dorsal Ant Cingulate				0.424	4.92	4.00	0.000	8	26	38
Occipital	0.000	613	0.000							
R Cuneus				0.341	5.18	4.15	0.000	10	−88	22
L Cuneus				0.436	4.83	3.95	0.000	−8	−86	22
R Calcarine				0.436	4.69	3.86	0.000	2	−86	14

Height threshold: T = 3.50, p = 0.001; Extent threshold: k = 300 voxels, p = 0.000.

Table S16: Voxel-Based Analysis: BOLD Response Regressed on Net Payoff

Cluster	p_{FDR}	k_E	p_{unc}							
Local Max				p_{FDR}	t	z	p_{unc}	x	y	z
R Caudate	0.000	479	0.000							
R Caudate				0.373	6.07	4.60	0.000	20	24	6
R Caudate				0.373	5.95	4.54	0.000	22	38	4
R Caudate				0.373	5.41	4.27	0.000	20	42	12
L Putamen	0.008	269	0.001							
L Putamen				0.373	5.81	4.47	0.000	−30	6	−6
L Putamen				0.601	4.61	3.81	0.000	−22	20	−2
L Putamen				0.601	4.54	3.77	0.000	−16	10	−4
Caudate	0.000	880	0.000							
L Caudate				0.373	5.49	4.31	0.000	−14	26	14
L Caudate				0.373	5.33	4.22	0.000	−4	20	12
R Caudate				0.373	5.22	4.17	0.000	6	28	10
R Caudate/Putamen	0.134	122	0.012							
R Caudate				0.636	4.45	3.72	0.000	26	4	30
R Caudate				0.636	4.35	3.66	0.000	40	−2	24
R Caudate				0.939	3.95	3.40	0.000	20	6	24

Height threshold: T = 3.50, p = 0.001; Extent threshold: k = 100 voxels, p = 0.020.

Table S17: Voxel-Based Analysis: Connectivity in the Resting Phase

Cluster	p_{FDR}	k_E	p_{unc}							
Local Max				p_{FDR}	t	z	p_{unc}	x	y	z
MPFC/Cingulate/R Inf Temporal	0.000	24153	0.000							
VMPFC				0.000	18.39	7.77	0.000	0	46	−6
Ant Cingulate				0.000	16.42	7.47	0.000	2	44	12
VMPFC				0.000	15.53	7.32	0.000	−6	44	−16
L Angular /Mid Temporal	0.000	2243	0.000							
L Angular Gyrus				0.000	15.29	7.28	0.000	−46	−66	30
L Mid Temporal				0.001	13.79	7.00	0.000	−56	−58	20
L Mid Temporal				0.001	13.59	6.96	0.000	−52	−66	22
R Angular Gyrus	0.000	1355	0.000							
R Angular Gyrus				0.005	11.86	6.57	0.000	42	−64	26
R Angular Gyrus				0.007	11.33	6.44	0.000	50	−66	30
R Angular Gyrus				0.013	10.45	6.21	0.000	54	−60	26
L Mid Temporal	0.000	1239	0.000							
L Mid Temporal				0.008	11.04	6.37	0.000	−58	−10	−16
L Mid Temporal				0.010	10.77	6.29	0.000	−62	−20	−18
L Mid Temporal				0.011	10.63	6.26	0.000	−60	−12	−6

Height threshold: T = 7.00, p = 0.000; Extent threshold: k = 300 voxels, p = 0.000.

Table S18: Voxel-Based Analysis: BOLD Response Regressed on Task minus Resting Phase

Cluster	p_{FDR}	k_E	p_{unc}							
Local Max				p_{FDR}	t	z	p_{unc}	x	y	z
Occipital/ Sup Parietal/ L Precentral	0.000	21707	0.000							
R Mid Occipital				0.000	14.02	7.04	0.000	30	−66	34
L Mid Occipital				0.000	13.68	6.97	0.000	−32	−88	12
R Mid Occipital				0.000	13.44	6.92	0.000	32	−72	24
Suplementary Motor	0.000	850	0.000							
L Suplementary Motor				0.000	9.01	5.77	0.000	−6	8	50
R Suplementary Motor				0.000	8.64	5.65	0.000	8	16	50
L Suplementary Motor				0.185	4.60	3.81	0.000	−8	−4	58
R Frontal/Precentral Gyrus	0.000	937	0.000							
R Mid Frontal				0.001	8.23	5.51	0.000	32	−2	54
R Sup Frontal				0.048	5.46	4.30	0.000	28	10	64
R Mid Frontal Gyrus	0.000	1044	0.000							
R Mid Frontal				0.001	7.66	5.29	0.000	46	34	34
R Mid Frontal				0.003	7.15	5.09	0.000	38	36	28
R Mid Frontal				0.006	6.77	4.93	0.000	36	56	−2

Height threshold: T = 3.50, p = 0.001; Extent threshold: k = 300 voxels, p = 0.000.

Table S19: Voxel-Based Analysis: BOLD Response Regressed on Resting Phase minus Task

Cluster	p_{FDR}	k_E	p_{unc}							
Local Max				p_{FDR}	t	z	p_{unc}	x	y	z
MPFC/Cingulate	0.000	10583	0.000							
VMPFC				0.000	11.60	6.51	0.000	0	58	−2
VMPFC				0.002	9.23	5.84	0.000	−6	38	−10
Posterior Cingulate				0.002	9.07	5.79	0.000	−8	−56	14
L Angular/Mid Occi+Temporal	0.000	756	0.000							
L Angular				0.001	10.12	6.11	0.000	−42	−74	40
L Mid Occipital				0.003	8.70	5.67	0.000	−46	−74	32
L Mid Temporal				0.013	6.74	4.92	0.000	−42	−64	22
L Insula/Supramaginal	0.000	6019	0.000							
L Insula				0.002	8.90	5.74	0.000	−30	12	−12
L Supramarginal				0.004	8.13	5.47	0.000	−60	−30	28
L Insula				0.005	7.90	5.38	0.000	−36	6	−12
R Angular/Sup Temporal	0.000	6235	0.000							
R Angular				0.004	8.15	5.48	0.000	54	−68	30
R Sup Temporal				0.007	7.56	5.25	0.000	52	−22	14
R Sup Temporal				0.008	7.33	5.16	0.000	58	−16	8
L Sup Frontal	0.000	482	0.000							
L Sup Frontal				0.018	6.42	4.77	0.000	−20	28	44
L Sup Frontal				0.020	6.31	4.72	0.000	−14	38	46
L Sup Frontal				0.043	5.67	4.41	0.000	−12	46	48

Height threshold: T = 3.50, p = 0.001; Extent threshold: k = 300 voxels, p = 0.000.

Table S20: ROI Analysis: Hidden States vs. Observed Payoffs Inference

Variable	*Estimate*	*Lower*	*Upper*	*SE*	*Df*	*t*	*p*
Fixed effect							
Medial Prefrontal	−0.145	−0.574	0.285	0.219	7344	−0.66	0.509
L Angular	0.034	−0.453	0.520	0.248	7344	0.14	0.892
R Angular	0.570*	0.075	1.064	0.252	7344	2.26	0.024
Prob (from states, M4)	1.287*	0.167	2.406	0.571	7344	2.25	0.024
Prob (from obs, M4a)	0.730	−0.740	2.201	0.750	7344	0.97	0.330
Random effect (SD)							
Medial Prefrontal	0.984	–	–	–	–	–	–
L Angular	1.095	–	–	–	–	–	–
R Angular	1.116	–	–	–	–	–	–
Prob (from states, M4)	1.478	–	–	–	–	–	–
Prob (from obs, M4a)	2.712	–	–	–	–	–	–
Error	3.400	–	–	–	–	–	–

*0 not included in the 95% Confidence Interval; Nbr. data = 7371 (resampling stage only);
The correlation between probabilities inferred from hidden states and observations was high (.92). However, the variation Inflation Factor was 6.5 for the inferrence from states and 6.6 for the inferrence from observations. Being smaller than 10, these values indicate that the high correlation had a limited impact on standard-errors. The p-values of the results are thus reliable.

References

1. Stephan K, Penny W, Daunizeau J, Moran R, Friston K (2009) Bayesian model selection for group studies. Neuroimage 46: 1004–1017.

2. Kriegeskorte N, Simmons W, Bellgowan P, Baker C (2009) Circular analysis in systems neuroscience–the dangers of double dipping. Nature Neuroscience 12: 535.

3. Brett M, Anton JL, Valabregue R, Poline JB (2002) Region of interest analysis using an SPM toolbox. Presented at the 8th International Conference on Functional Mapping of the Human Brain, June 2-6, Sendai, Japan.

4. R Development Core Team (2010) R: A Language and Environment for Statistical Computing. R Foundation for Statistical Computing, Vienna, Austria. URL `http://www.r-project.org`.

5. Weissenbacher A, Kasess C, Gerstl F, Lanzenberger R, Moser E, et al. (2009) Correlations and anticorrelations in resting-state functional connectivity MRI: A quantitative comparison of preprocessing strategies. Neuroimage 47: 1408–1416.
